# Lactuchelins represent lipopeptide siderophores produced by *Pseudomonas lactucae* that inhibit *Xanthomonas campestris*

**DOI:** 10.1093/ismejo/wrag003

**Published:** 2026-01-16

**Authors:** Guillaume Chesneau, Alba Noel, Dimitri Bréard, Alice Boulanger, Martial Briand, Sophie Bonneau, Chrystelle Brin, Marion Fischer-Le Saux, Yujia Liu, Andrew Hendrickson, Torben Nielsen, Alain Sarniguet, David Guilet, Adam Arkin, Lauren Lui, Matthieu Barret

**Affiliations:** Univ Angers, Institut Agro, INRAE, IRHS, SFR QUASAV, Angers F-49000, France; Department of Plant Microbe Interactions, Max Planck Institute for Plant Breeding Research, Cologne 50829, Germany; Univ Angers, Institut Agro, INRAE, IRHS, SFR QUASAV, Angers F-49000, France; Univ Angers, SONAS, SFR QUASAV, Angers F-49000, France; Laboratoire des Interactions Plantes-Microbes-Environnement (LIPME), Université de Toulouse, INRAE, CNRS, Castanet-Tolosan, France; Univ Angers, Institut Agro, INRAE, IRHS, SFR QUASAV, Angers F-49000, France; Univ Angers, Institut Agro, INRAE, IRHS, SFR QUASAV, Angers F-49000, France; Univ Angers, Institut Agro, INRAE, IRHS, SFR QUASAV, Angers F-49000, France; Univ Angers, Institut Agro, INRAE, IRHS, SFR QUASAV, Angers F-49000, France; Division of Environmental Genomics and Systems Biology, Lawrence Berkeley National Laboratory, Berkeley, CA 94720, United States of America; Division of Environmental Genomics and Systems Biology, Lawrence Berkeley National Laboratory, Berkeley, CA 94720, United States of America; Division of Environmental Genomics and Systems Biology, Lawrence Berkeley National Laboratory, Berkeley, CA 94720, United States of America; Univ Angers, Institut Agro, INRAE, IRHS, SFR QUASAV, Angers F-49000, France; Univ Angers, SONAS, SFR QUASAV, Angers F-49000, France; Division of Environmental Genomics and Systems Biology, Lawrence Berkeley National Laboratory, Berkeley, CA 94720, United States of America; Department of Bioengineering, University of California, Berkeley, CA 94720, United States; Division of Environmental Genomics and Systems Biology, Lawrence Berkeley National Laboratory, Berkeley, CA 94720, United States of America; Univ Angers, Institut Agro, INRAE, IRHS, SFR QUASAV, Angers F-49000, France

**Keywords:** Siderophore, *Xanthomonas campestris* pv. *campestris*, *Pseudomonas*, Intermicrobial Competition, Seed Microbiota, Lactuchelins

## Abstract

The seed is a habitat with limited resources and space. Although it is widely accepted that microbial competition is a key driver of the assembly of seed-associated microbial communities, the underlying mechanisms of this competition are not well understood. The initial objective of this work was to assess the importance of contact-independent microbial competition between the phytopathogenic bacterium *Xanthomonas campestris* pv. *campestris* 8004 (Xcc8004) and 30 strains representative of the bacterial populations most commonly associated with radish (*Raphanus sativus*) seeds. We identified *Pseudomonas lactucae* CFBP 13502 as a potent inhibitor of Xcc8004, mediated by exometabolites, specifically induced by certain seed-borne strains. Transcriptomic analysis linked this inducible activity to the upregulation of a gene cluster encoding a lipopeptide siderophore. Targeted gene deletion in *P. lactucae* CFBP 13502 confirmed that this cluster is essential for antagonism against Xcc8004. Furthermore, iron supplementation abolished this inhibitory effect, strongly supporting iron chelation as the underlying mechanism. Through comparative metabolomics, we elucidated the structure of a family of lipopeptide siderophores, produced by *P. lactucae* CFBP 13502, which we named lactuchelins. Our findings provide molecular evidence of competitive exclusion mechanisms at the seed microbiome interface, highlighting lactuchelins as a promising avenue for the development of seed-based biocontrol strategies against seed-borne phytopathogens.

## Graphical Abstract

**Graphical Abstract:**
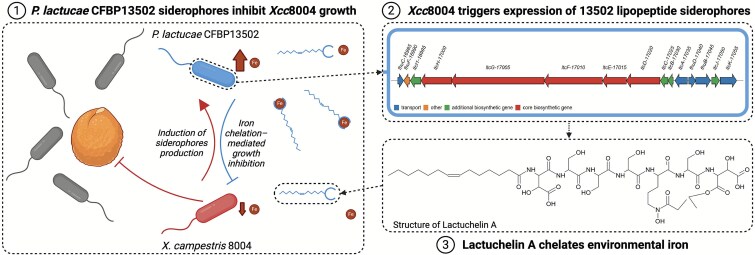

## Introduction

Historically, seeds have been viewed as a means of plant pathogens dispersal which can lead to the emergence of diseases in new geographical areas [1]. However, seed microbiota is not restricted to plant pathogens as seeds harbor diverse microbial communities [2] that can contribute to plant growth and disease suppression [3, 4]. Promoting these beneficial microbes at the expense of plant pathogens through seed microbiota engineering has recently emerged as a promising strategy [5]. However, this requires an understanding of the molecular mechanisms involved in intermicrobial competition within this habitat.

Due to their small size and limited nutrient reserves, seeds host microbial communities dominated by a few taxa [6, 7]. These constraints amplify priority effects, as early-arriving microbes pre-empt nutrients and limit the success of late colonizers [8]. Consequently, exploitative competition (i.e. efficient uptake and use of resources [9]) is a significant driver of community assembly during seed transmission [10, 11].

Although carbon is often considered the primary limiting nutrient in seeds, micro-elements availability presents also a significant challenge for microbial establishment. For instance, iron plays an important role in *Pseudomonas putida* establishment on seeds [12]. The ability to efficiently acquire iron can thus be a major determinant of microbial success during seed transmission. Iron acquisition is usually achieved through the secretion of siderophores, secondary metabolites that possess different iron-chelating functional groups such as catechols, hydroxamates, carboxylates, or phenolates [13]. Siderophores are well-known for their role in microbe-microbe interactions and competition [14].

Pyoverdines are a well-characterized group of siderophores produced by *Pseudomonas* species that contribute to their fluorescence property [15]. Pyoverdine biosynthesis genes are highly conserved, present in 97% of sequenced *Pseudomonas* genomes [16]. Non-fluorescent pseudomonads can also produce other siderophores such as corrugatin [17] and its derivatives ornicorrugatin and histicorrugatin [18]. These peptidic siderophores are acylated with a lipid chain of variable length. Lipopeptide siderophores are highly prevalent in marine bacteria, because the amphiphilic properties of these molecules could limit siderophore diffusion via membrane attachment [19–22]. Lipopeptide siderophores are also present in a number of plant-associated bacteria, such as strains of the *Burkholderia cepacia* group (ornibactins [23]), *Cupriavidus taiwanensis* (taiwachelins [24]), *Herbaspirillum seropedicae* (serobactins [25]), *Variovorax boronicumulans* (variochelins [26]), or *Azotobacter chroococcum* (crochelins [27]).

Despite the importance of iron acquisition in microbial competition, the role of siderophore-mediated interactions in seed-borne microbial communities remains underexplored. By analyzing a set of seed-borne bacterial strains that are consistently seed transmitted [10, 28], we identified a non-fluorescent strain belonging to the *Pseudomonas lactucae* species [29] that significantly reduced the growth of *X. campestris* pv. *campestris* 8004 (Xcc8004). Compounds responsible for this activity are related to a new lipopeptide siderophore family that we propose to name lactuchelins. Lactuchelins production in *P. lactucae* CFBP 13502 is enhanced during interactions with specific bacterial strains including Xcc8004. The gene cluster involved in the biosynthesis of this compound is distributed in 10 species of *Pseudomonas* including several strains isolated from seed. Investigating how seed-associated bacteria utilize these siderophores to outcompete plant pathogens could provide strategies for microbiome-based disease management in crops.

## Materials and Methods

### Bacterial strains and culture media

Thirty bacterial strains covering the diversity of seed microbial communities [2] were selected from a collection of 530 strains (Table S1). All these strains were isolated from radish seed samples (*Raphanus sativus* var. Flamboyant5) collected at the Experimental station of the National Federation of Seed Multipliers (FNAMS, 47°28012.42 N-0°2344.30 W, Brain-sur-l’Authion, France) in 2013, 2014, and 2015 [30] except one strain isolated from radish flower in 2016 [31]. These strains were either isolated from unsterilised seed lots or surface sterilised (10 g, ~1000 seeds) using the following protocol: 1 min sonication (40 Hertz), soaking for 1 min in 96° ethanol, 5 min in 2.6% sodium hypochlorite, 30 s in 96° ethanol, and rinsed 3 times with sterile water. Bacterial strains were recovered from 1/10 strength Tryptic Soy Agar (17 g.l^−1^ tryptone, 3 g.l^−1^ soybean peptone, 2.5 g.l^−1^ glucose, 5 g.l^−1^ NaCl, 5 g.l^−1^ K2HPO4, and 15 g.l^−1^ agar) after 7 days of incubation at 18°C. These isolates were typed through sequencing of a portion of *gyrB* with the primer set gyrB_aF64 and gyrB_aR353 following the procedure described earlier [32].

The strain CFBP 13502 has been renamed *Pseudomonas lactucae* following its taxonomic identity reassessment. Detailed information about the taxonomic affiliation reassessment can be found in Supplementary methods.

*X. campestris* pv. *campestris* 8004 (Xcc8004) is a spontaneous rifampicin-resistant strain [33], which derives from *X. campestris* pv. *campestris* NCPPB 1145. Xcc, the causal agent of black rot disease, is a major bacterial pathogen of Brassica crops such as cauliflower, cabbage, mustard, and radish [34]. This seed-transmitted pathogen has a complex lifestyle involving both epiphytic and endophytic stages [34].

All strains were routinely grown in 1/10 strength Tryptic Soy Broth (TSB 10%) at 28°C, supplemented when needed with 15 g. l^−1^ of agar and 50 μg.ml^−1^ of rifamycin.

Mono-culture and co-culture of the 30 selected bacterial strains with Xcc8004 were performed at 28°C under constant shaking (150 rpm) during 24 h using a starting OD_600_ of 0.02. Culture supernatant was filtered through 0.22 μm filters. The resulting cell-free supernatant (CFS) was mixed 40% (v/v) with 50% (v/v) of 1/5 strength TSB and 10% (v/v) of a fresh cell suspension of Xcc8004 at an initial OD_600_ of 0.2. Two hundred μl of the resulting mix was added per well of 96 well-plates. OD was recorded every hour during 24 h, under constant shaking (150 rpm) at 28°C (Fig. S1). Iron supplementation was performed by adding a range of FeCl_3_ concentration (from 0.3 μM to 50 μM final concentration) in the coculture or in the CFS. The areas under the curve (AUC) were calculated using library growthcurver R package (version 0.3.1).

Impact of lactuchelins on Xcc8004 seedling transmission was evaluated by inoculated radish seeds (*R. sativus* var. Flamboyant5) with *P. lactucae* CFBP 13502 wild-type or the isogenic lactuchelins-deficient mutant (see section Construction of *P. lactucae* CFBP 13502Δ*ltcJ*). Both strains (wt and mutant) were seed-inoculated with Xcc8004 at a 1:1 ratio using a concentration of 10^8^ cells·ml^−1^ from 24 h TSA10% plates. Fifty seeds of each treatment were placed in sterile plastic boxes containing blotter paper supplemented with 25 ml of sterile water. Boxes were placed in a growth chamber (photoperiod: 16 h/8 h, 20°C). Seedling (corresponding to the emergence of the cotyledons) were collected 60 h post-inoculation and placed in sterile bags containing 10 ml of sterile water. Samples were serial diluted and plated on TSA 10 with 50 μg.ml^−1^ of rifamycin (Xcc8004) or kanamycin (CFBP13502 wt and mutant). The number of colony forming units were recorded after 48 h at 28°C. Three independent biological, each composed of three technical replicates (one box per technical replicate) were performed for each co-inoculation.

### Genome sequencing and annotation

Genome sequences of radish-associated bacterial strains were initially obtained on a HiSeq 4000 PE150 System (Illumina) [10]. In addition, DNA of *P. lactucae* CFBP 13502 was subjected to long-read sequencing (PRJNA454856). High-molecular weight (HMW) DNA was extracted using the Masterpure kit (Lucigen Corporation, USA) with slight modifications. Detailed information about total DNA extraction can be found in Supplementary methods. Nanopore libraries were created using the SQK-LSK109 kit and sequenced on a MinION (Oxford Nanopore Technologies, UK) with a R9.4.1 flow cell. Basecalling, adapter removal, and barcode removal of the nanopore reads was done with Guppy 4.0 software (Oxford Nanopore Technologies, UK). Illumina reads previously obtained [10] were trimmed and cleaned using BBtools [35]. Illumina and nanopore reads were used as input to Unicycler [36] for hybrid genome assembly. Unicycler was run with default parameters. Visualization of the lactuchelin gene cluster was performed with geneviewer 0.1.10 [37].

### RNA-Seq experiments

Transcriptome analysis of *P. lactucae* CFBP 13502 (hereafter referred to as CFBP 13502) was performed during co-culture with two strains (*Stenotrophomonas rhizophila* CFBP 13503 and Xcc8004) that produced a CFS with bacCterial growth inhibition potential. These transcriptomes profiles were compared to co-culture of CFBP 13502 with two strains (*Pantoea agglomerans* CFBP 13505 and *Pseudomonas viridiflava* CFBP 13507) that did not trigger CFS with Xcc8004 inhibitory activity. Four independent replicates were performed per co-culture at a starting OD_600_ = 0.02. Total RNA of co-cultures was isolated by a modification of the method of [38]. Detailed information about total RNA extraction can be found in Supplementary methods. RNA-Seq libraries were prepared with the QIAseq FastSelect -5S/16S/23S and QIAseq stranded total RNA Lib kits following manufacturer’s instructions (Qiagen, Venlo, Netherlands). RNA fragmentation was performed at 89°C for 6 min. The quality of the resulting libraries was assessed with a Bioanalyzer (Agilent, Santa Clara, CA) and concentration of libraries and subsequent equimolar pool was quantified with quantitative PCR using Illumina primers (Roche, Basel, Switzerland). The equimolar pool was sequenced on a NextSeq 550 System (Illumina) using High Output 150 cycles cartridge.

A median of 25 million paired-end reads (min = 16, max = 32) was obtained per sample. Quality and adapter trimming was performed with Trim Galore 0.6.3 (https://github.com/FelixKrueger/TrimGalore) using a Phred score of 20. Reads were mapped on CFBP 13502 genome sequence with Salmon [39]. Proteins function were inferred with PaperBLAST [40]. Iron gene repertoire of CFBP 13502 was estimated with FeGenie [41].

### Genome-wide mutant fitness assays

RB-TnSeq libraries of CFBP 13502 (created using mariner transposons following the protocol of [42]) and *X. campestris* pv. *campestris* 8004 [43] were used in this study. CFBP 13502 library was screened for alteration in siderophore production on CAS agar plates. A single aliquot of CFBP 13502 mutant library was inoculated in LB supplemented with kanamycin (50 mg.ml^−1^) and grown overnight at 28°C under constant shaking. The resulting suspension was serial-diluted and plated on LB media supplemented with kanamycin (50 mg.ml^−1^). Approximately 10 000 colonies were transferred to Chrome Azurol S (CAS) agar plates. CAS agar plates were prepared following the protocol of [44]. Detailed information about CAS agar plates preparation can be found in Supplementary methods. After 12 h of incubation at 28°C, an orange halo appeared around the bacterial colonies, which corresponds to the production of a siderophore. Colonies that did not form this halo were sequenced with the primer pairs U1_fwd/U2_rev (Table S2) that target the U1/U2 priming sites [42]. PCR products were sequenced and reads were mapped to the CFBP 13502 genome sequence using the position of barcodes.

The Xcc8004 mutant library was employed for running pooled mutant fitness assays [42, 45]. Detailed information about pooled mutant fitness assays can be found in Supplementary methods. We incorporated the Xcc8004 mutant library (starting OD_600_ = 0.02) in Xcc8004 and CFBP 13502 cell-free supernatant supplemented with TSB20, and compared, as controls, the fitness of the library in three other cell-free supernatant: the mutant library grown (i) in PBS, (ii) in Xcc8004 cell-free supernatant, and (iii) in CFBP 13502 cell-free supernatants (Cultures were performed following 1/4/5 ratio described in “Phenotype screening for Xcc8004 inhibition” section). We collected the mutant population in our various conditions after 24 h of growth, and extracted genomic DNA from the Time0 and condition samples using NucleoSpin 96 Food kit (Macherey-Nagel) following the supplier’s recommendations. Detailed information about DNA extraction can be found in Supplementary methods.

To analyze our data, we used a pipeline developed to acquire mutant fitness scores from raw sequencing data. Detailed information about the pipeline can be found in Supplementary methods. To infer the function of Xcc8004 genes based on mutant phenotype, we used the SEED database from the fitness browser (fit.genomics.lbl.gov). We also inferred gene function based on scientific articles published using PaperBLAST [40].

### Construction of *P. lactucae* CFBP 13502ΔltcJ

Deletion of *ltcJ* (APEGCL_17050) was performed by allelic exchange using the suicide vector pEX18Tc [46]. The deletion plasmid pEX18Tc-Δ*ltcJ* was constructed using the TEDA cloning procedure [47]. Detailed Information about the TEDA cloning procedure can be found in Supplementary methods. Plasmids were transferred to CFBP 13502 by conjugation. CFBP 13502 transconjugants were selected on TSA10 supplemented with tetracycline (20 μg.ml^−1^). The resulting colonies were grown in TSB10% (28°C, 120 rpm, 3 h) and bacterial suspensions were spread on TSA10 supplemented with 5% saccharose. Allelic exchanges were validated by tetracycline resistance loss, PCR, and sequencing.

Reintroduction of the *ltcJ* gene in CFBP 13502Δ*ltcJ* was performed with the pUC18T-miniTn7-PlppGFP-KmR plasmid (obtained from Mette Burmølle, University of Copenhagen) for cis-complementation of Δ*ltcJ*. This plasmid was digested with SmaI and assembled by HiFi assembly with the PCR-amplified product of *ltcJ* obtained from CFBP 13502 with the Q5 High-Fidelity DNA polymerase and the primer pairs listed in Table S2. Plasmids were extracted with the NucleoSpin plasmid kit (Macherey-Nagel), and insertion regions were verified by sequencing. *Escherichia coli* MFDpir [48] was transformed with pUC18T-miniTn7-plppGFP-kmR-*ltcJ*. Triparental mating conjugation was achieved with CFBP 13502Δ*ltcJ*, MFDpir-pUC18T-miniTn7-PlppGFP-KmR-*ItcJ*, and MFDpir-pTNS2 [49]. CFBP 13502Δ*ltcJ* transconjugants were selected on TSA10 supplemented with kanamycin (50 μg.ml^−1^). The resulting colonies were spread on TSA10 (28°C, 48 h) and the presence of the *ltcJ* gene was validated by PCR.

### Comparative metabolic profiling

To identify the metabolite inhibiting Xcc8004 growth we performed comparative metabolic profiling of Xcc8004 + CFBP 13502 CFS, Xcc8004 CFS, CFBP 13502 CFS, and TSB CFS. Xcc8004 + CFBP 13502 CFS was freeze-dried and submitted to a liquid/liquid extraction with H2O *versus* four different organic solvents, ethyl acetate (AcOEt), butanol (BuOH), dichloromethane (DCM), and methyl *tert*-butyl ether (MtBE). Each extract was evaporated then tested against Xcc8004 (section In vitro growth inhibition test). BuOH extract was selected due to its inhibitory effect on Xcc8004 growth. Liquid/liquid extraction (H2O/BuOH) was performed on the four cell-free supernatants and each fraction was analyzed with an UPLC-HRMS/MS at a concentration of 100 μg.ml^−1^. Detailed information about the metabolomic analysis by Ultra High Liquid Chromatography and mass spectrometry (UPLC-HRMS/MS) can be found in Supplementary methods.

### Culture, extraction, purification, and identification of lactuchelins

Bulk extraction of the metabolites was performed on liquid cultures of CFBP 13502 for 24 h in 2 l of iron-depleted M9 medium at 28°C. Subsequently, cells were removed from the supernatant by centrifugation (10 min at 5000 G) and filtration through vacuum filtration system with 0.22 μm filter membrane (TPP, Switzerland).

The resulting CFS was then subjected to freeze-drying (22.5 g), redissolved in 1 l of water complemented with iron chloride and extracted 3 times against BuOH (3 × 1 l). The resulting aqueous (LLW) and BuOH (LLB) fractions were concentrated *in vacuo* at 40°C.

The LLB fraction (1.1 g) was absorbed on Polygoprep C18 bulk media (50–60 μm) with a ratio media/fraction of 2/1, and transferred into a pre-conditioned HyperSep C18 cartridge (5000 mg; 40–60 μm). A ratio bed weight/fraction of 20/1 was applied. The adsorbed metabolites were then eluted with 50 ml of 3 step solvents: water (SW), water/MeOH (50/50; SWM), and MeOH (SM). The resulting fractions were concentrated *in vacuo* at 40°C. Finally, SWM (482 mg) was separated on a preparative HPLC system (Supplementary method), leading to five fractions (F1 to F5) which were then concentrated *in vacuo* at 40°C.

F4 (18.4 mg) was subjected to an iron removal protocol. Ferric iron was removed according to literature protocols [23] and replaced by Gallium for NMR analysis (Supplementary methods). A final purification of the Ga(III)-F4 fraction (12 mg) was accomplished following the previously established preparative purification method to remove excess of gallium and other impurities. All NMR spectroscopy experiments (1H, 13C, and 2D) of lactuchelin (6.5 mg) were recorded as the Ga(III) adduct in DMSO-*d6* on a Bruker Avance NEO 600 MHz spectrometer equipped with a 5 mm DCH cryoprobe. HRMS fragmentation pattern was recorded during the comparative metabolic profiling.

### *In vitro* growth inhibition assay against *X. campestris* pv. campestris 8004

The inhibition activity of fractions on Xcc8004 growth was evaluated by an in vitro growth inhibition assay. Fractions were resuspended at 1 mg.ml^−1^ in a 9:1 (v/v) water/DMSO mixture and mixed with 50% (v/v) of a fresh cell suspension of Xcc8004 at an initial concentration of 1600 CFU.μl^−1^ in TSB 20%. One hundred μl of the resulting mix was added per well of 96 well-plates. These generation plates were placed in a millifluidics system (MilliDrop Azurevo, Gold Standard Diagnostics MilliDrop, France) system was used to assess the effect of fractions on the growth curve of Xcc8004 grown in TSB 10%_._ From the wells, the automated system generates a train of 1 μl droplets separated by air bubbles and moving in a carrier fluid that was incubated at 28°C for 30 h. The bacterial growth within each droplet was assessed by light scattering measurements at different timepoints. The 96 well-plates used for the generation of droplets were also incubated in a microplate spectrophotometer at 28°C and the OD was recorded every hour during 24 h, under constant shaking (150 rpm).

### Statistical analyses

*AUC*. Comparisons between samples were analyzed using t-test. Effect sizes were calculated using Hedges’ g. Hedges’ g > 2 were considered as a large effect size.

*RNASeq*. Differentially-expressed genes (DEGs) were determined using DeSeq2 v1.36 [50] at an adjusted *P* value <0.01 (Wald Test) and a logarithmic fold change (|log_2_FC| > 2) estimated with apeglm v1.18 [51].

*RB-TnSeq*. Differences in gene fitness values were estimated with a moderated t statistic [42]. Genes with |fitness| > 1 and |t| > 5 were considered as significantly altered.

## Results

### Cell-free supernatant from *P. lactucae* CFBP 13502 inhibits Xcc8004 growth

**Figure 1:**
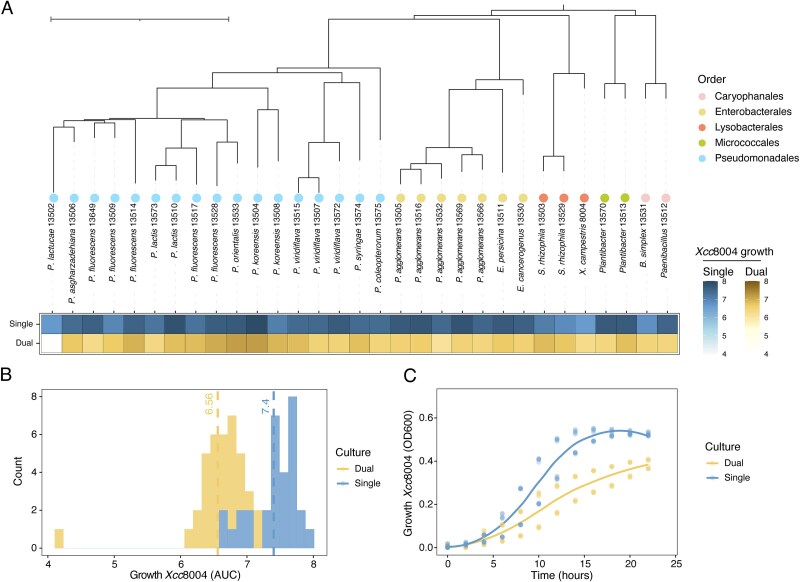
Cell-free supernatant (CFS) from *P. lactucae* CFBP 13502 inhibits *Xanthomonas campestris* pv. *campestris* 8004 (Xcc8004) growth in TSB10%. (A) Phylogenetic analysis of the 30 seed-borne bacterial strains employed in this work. Heatmap displays Xcc8004 growth (Area under the curve, AUC) following supplementation of CFSs derived from pure cultures of 30 seed-borne bacterial strains (blue-single) or co-culture with Xcc8004 (yellow-dual). n = 3 replicates. (B) Distribution of Xcc8004 growth (AUC) after supplementation with CFSs from pure culture (blue-single) or co-culture (yellow-dual). (C) Representative growth curves of Xcc8004 supplemented with CFS of a pure culture of *P. lactucae* CFBP 13502 (blue—single) or a co-culture of *P. lactucae* CFBP 13502 and Xcc8004 (yellow—dual). n = 3 replicates.

The growth of the phytopathogenic strain *X. campestris* pv. *campestris* 8004 (Xcc8004) was evaluated in TSB10% medium supplemented with various cell-free supernatants (CFSs) (Fig. S1). These CFSs were derived from (*i*) pure overnight cultures of 30 seed-borne bacterial strains or from (*ii*) co-cultures of each bacterial strain with Xcc8004 (Fig. 1A and Table S1).

Most of the CFSs derived from single or co-cultures did not modulate Xcc8004 growth (Fig. 1A and B and Fig. S2). The areas under the curve (AUC) of Xcc8004 following supplementation of CFSs collected from single or co-cultures were overall similar, with average AUC values of 7.4 and 6.6, respectively (Fig. 1B). However, the CFS collected from the co-culture of *P. lactucae* CFBP 13502 and Xcc8004 decreased the AUC of Xcc8004 to 4.2 (Fig. 1B), which represented a ~40% reduction compared to the CFS of *P. lactucae* CFBP 13502 alone (Fig. 1C). More specifically, CFS from this coculture decreased the growth rate (0.31 h^−1^, 35% reduction) and maximal population density of Xcc8004(0.38, 30% reduction), while increased the time to reach half of the maximum density by 2 h (Fig. S2). Altogether these results suggest that the co-culture of *P. lactucae* CFBP 13502 with Xcc8004 produced metabolite(s) limiting Xcc8004 growth.

To determine whether the growth reduction effect observed was specific to Xcc8004 or could be generalized to other bacterial strains, (*i*) CFS collected from a pure culture of *P. lactucae* CFBP 13502 and (*ii*) CFS collected from the coculture of *P. lactucae* CFBP 13502 and Xcc8004 were applied to cultures of the 30 bacterial strains employed in this study. The growth of 28 strains was inhibited by the CFS derived from the co-culture in comparison with the CFS from the pure culture (Fig. S3). However, the magnitude of this inhibitory effect varied, with reductions ranging from 15 to 60% of their AUC. The three strains that were not sensitive to the co-culture supernatant were affiliated to *P. asgharzadehiana* CFBP 13506, *P. coleopterorum* CFBP 13575, and *P. lactucae* CFBP 13502.

### Transcriptome profiling of *P. lactucae* CFBP 13502 reveals induction of a biosynthetic gene cluster encoding a lipopeptide siderophore

**Figure 2:**
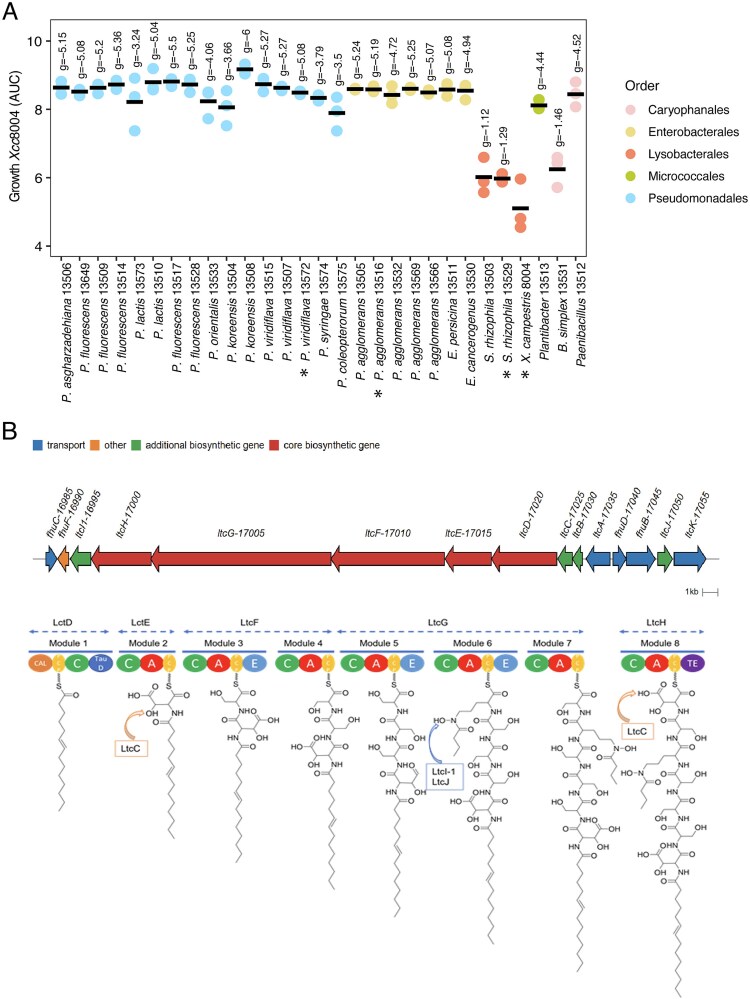
Activation of a lipopetide siderophore biosynthetic gene cluster in *P. lactucae* CFBP 13502 during competitive interactions with Xcc8004 and seed-borne bacteria. (A) Area under the curve of Xcc8004 growth following supplementation of CFSs derived from co-culture of *P. lactucae* CFBP 13502 and 30 seed-borne bacterial strains. n = 3 replicates. Bacterial strains are colored according to their taxonomic affiliation at the order level. Effect sizes for each supernatant relative to the control supernatant (Xcc8004) were calculated using hedges’ g. values of hedges’ g (shown above each strain) > 2 were considered as a large effect size. Stars indicated the cocultures employed for RNASeq experiment. n = 4 replicates. (B) Biosynthetic gene cluster involved in synthesis of a lipopeptide siderophore and *in silico* prediction of lactuchelin structure. (top) Composition of the biosynthetic gene cluster containing 5 NRPS genes. (bottom) Domain architecture of the NRPS proteins and amino acids predicted to be loaded, CAL: Co-enzyme A ligase domain, PCP: Peptidyl-carrier protein domain, C: Condensation domain, TauD: hydroxylase, A: Adenylation domain, E: Epimerization domain, TE: Thioesterase domain.

To test whether the observed Xcc8004 growth reduction was also produced by *P. lactucae* CFBP 13502 during interactions with other bacterial strains, CFSs were collected from co-cultures between *P. lactucae* CFBP 13502 and the 30 remaining bacterial strains. A decrease in the AUC of Xcc8004 growth was observed for four of these dual CFSs derived from co-cultures of *P. lactucae* CFBP 13502 with (*i*) Xcc8004, (*ii*) *S. rhizophila* CFBP 13503, (*iii*) *S. rhizophila* CFBP 13529, and (*iv*) *Bacillus simplex* CFBP 13531 (Fig. 2A).

Transcriptome profiling of *P. lactucae* CFBP 13502 was performed in two co-cultures that produced CFSs with Xcc8004 growth inhibition (*P. lactucae* CFBP 13502 with *S. rhizophila* CFBP 13503 or Xcc8004) and two co-cultures without reduction of Xcc8004 growth (*P. lactucae* CFBP 13502 with *P. agglomerans* CFBP 13505 or *P. viridiflava* CFBP 13507, Fig. 2A). RNAs were collected after 6 h of co-culture, which corresponded to the late exponential growth phase of *P. lactucae* CFBP 13502.

Expression was detected in 97% of the 5687 predicted genes of *P. lactucae* CFBP 13502. Of the 99 differentially expressed genes (DEGs, *P <* .01, |log_2_FC| > 2), 29 were associated with iron acquisition and iron gene regulation (Table S3). Among these 29 up-regulated genes, 12 were located within a biosynthetic gene cluster (BGC) over a 50 kb region (Fig. 2B). According to antiSMASH v7.0 [52], this BGC possesses sequence similarities to BGC associated with lipopeptide siderophores. The BGC of *P. lactucae* CFBP 13502 is composed of five non-ribosomal peptide synthases (*ltcD-H*) involved in the potential incorporation of the acyl chain and seven amino acids (Fig. 2B). Two of these amino acids, aspartate and ornithine, are likely modified by an aspartate β-hydoxylase (*ltcC*, [53]), a L-ornithine N(5)-monooxygenase (*ltcI_1*), and a N-hydroxyornithine acetyltransferase (*ltcJ*). The BGC also encoded a thioesterase (*ltcB*) and an ABC transporter (*ltcA*) putatively involved in translocation across the cytoplasmic membrane into the periplasm. The uptake of the ferrisiderophore in the periplasm is probably mediated through a specific TonB-dependent transporter (*ltcK*) and translocation across the inner membrane is likely performed through the ABC transporter FhuBCD. Iron removal is then probably performed through FhuF. Of note *fhuC*, *fhuD,* and *fhuB* were the only genes not differentially expressed under our experimental conditions. Moreover, other genes potentially involved in siderophore production were located outside the BGC. This is the case for a small MbtH like protein (APEGCL_19865 [54]), and a protein-coding gene *ltcI_2* (APEGCL_18425) sharing 72% of identity at the nucleic acid level with *ltcI_1*. According to these genomic predictions, the BGC of *P. lactucae* CFBP 13502 is therefore potentially involved in the production of a lipopeptide siderophore. We propose to name this siderophore lactuchelin.

The distribution of the lactuchelin BGC was searched in the 52 282 *Pseudomonas* genomic sequences available at the time of analysis (May 2025). According to protein similarity search this gene cluster is present in 53 *Pseudomonas* strains (Table S4). These strains are affiliated to 10 bacterial species, which belonged to *P. fluorescens*, *P. putida,* and *P. syringae* groups.

### Lactuchelin mediates inhibitory activity of *P. lactucae* CFBP 13502 against Xcc8004

**Figure 3:**
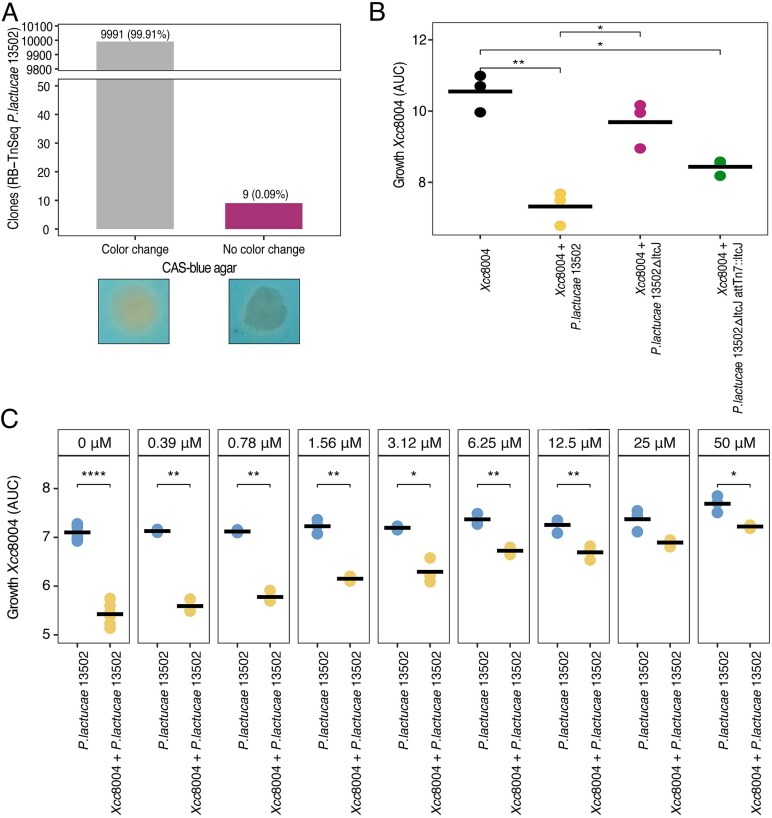
Siderophore mutants and iron rescue confirm *P. lactucae* CFBP 13502 inhibits Xcc8004 *via* lactuchelin production. (A) RB-TnSeq *P. lactucae* CFBP 13502 clones (n = 10 000) siderophore-production was monitored through color change on CAS medium. Representative images of the observed phenotypes are shown below. (B) Xcc8004 growth was monitored in TSB10% medium supplemented with CFSs from Xcc8004 (black) and co-cultures of Xcc8004 and *P. lactucae* CFBP 13502 (yellow), *P. lactucae* CFBP 13502Δ*ltcJ* (purple), and *P. lactucae* CFBP 13502Δ*ltcJ att*Tn7*::ltcJ* (green). Stars denote significance (pairwise t-tests with Holm correction for multiple comparisons). n = 3 replicates. (C) Xcc8004 growth was monitored in TSB10% medium supplemented with CFSs from *P. lactucae* CFBP 13502 (blue) and co-culture of Xcc8004 and *P. lactucae* CFBP 13502 (yellow). Each CFS was supplemented with various concentrations of FeCl_3_ ranging from 0.39 μM to 50 μM. Stars denote significance (pairwise t-tests with Holm correction for multiple comparisons). n = 3 replicates.

To validate that the lactuchelin BGC is involved in siderophore production, a screening of a RB-TnSeq library of *P. lactucae* CFBP 13502 was performed on CAS plates. Based on this screening, nine clones (out of 10 000) were not able to induce color changes, which suggest that they no longer produced siderophores (Fig. 3A). According to the sequencing, eight different barcodes were inserted in five different CDSs (Table 1). Four of these CDSs were located in the lactuchelin BGC (*ltcD*; *ltcE*, *ltcG*, and *ltcJ*) whereas the last CDS corresponds to the PvdS transcriptional regulator.

**Table 1:** RB-TnSeq mutant candidates within the lactuchelin biosynthetic gene cluster.

Barcode	n	Strand	Position	Locus tag	Annotation
TTAACAAAGTGTAAGCCCCG	1	+	3 925 529	APEGCL_17005	Non-ribosomal peptide synthase LtcG
TGAATCCACACGCCACCAGA	1	−	3 926 247	APEGCL_17005	Non-ribosomal peptide synthase LtcG
CACCCGTTCGTCCCCCCCCC	1	−	3 942 457	APEGCL_17015	Non-ribosomal peptide synthase LtcE
GCTCCCCTCGAACCCGCCCA	1	+	3 944 692	APEGCL_17020	Non-ribosomal peptide synthase LtcD
GTTATTTTTGCAGGGTGGT	2	−	3 948 140	APEGCL_17020	Non-ribosomal peptide synthase LtcD
ACTCATTGACTCTGGCCTTA	1	−	3 948 140	APEGCL_17020	Non-ribosomal peptide synthase LtcD
CTCTCCACCTGCACCAGTAT	1	−	3 956 238	APEGCL_17050	N-hydroxyornithine acetyltransferase LtcJ
ACGTCACAATTTCACTCAGA	1	+	4 565 287	APEGCL_19915	Sigma factor PvdS

The table lists unique barcodes for each mutant (Barcode), the number of clones detected with each barcode (n), the strand orientation of the insertion (Strand), genomic position of the transposon insertion (Position), associated locus tag (Locus tag), and the predicted gene function (Annotation).

To verify data obtained with insertion mutants, a deletion mutant of *ltcJ* was generated by allele exchange (hereafter *P. lactucae* CFBP 13502Δ*ltcJ*). In addition chromosomal complementation with a full copy of *ltcJ* was introduced at the *att*Tn7 site with the plasmid pUC18T-miniTn7-plppGFP-KmR-*ltcJ* (hereafter *P. lactucae* CFBP 13502Δ*ltcJ att*Tn7*::ltcJ*). Finally, Tn7 transposition of the restricted plppGFP-KmR cassette was also performed in *P. lactucae* CFBP 13502 (hereafter *P. lactucae* CFBP 13502 *att*Tn7). Whereas *P. lactucae* CFBP 13502 and *P. lactucae* CFBP 13502 *att*Tn7 produced an orange halo on CAS plates, the deletion mutant *P. lactucae* CFBP 13502Δ*ltcJ* was no longer able to displace the CAS dye. The phenotype was restored through complementation with a full version of *ltcJ* (Fig. S4). Overall, these results showed that lactuchelin is a siderophore.

To confirm that lactuchelin was involved in growth reduction of Xcc8004, *P. lactucae* CFBP 13502, CFBP 13502Δ*ltcJ*, *and* CFBP 13502Δ*ltcJ att*Tn7*::ltcJ* were cultured overnight in M9 minimal media without iron supplementation. The corresponding CFSs were added in TSB10% medium and the growth of Xcc8004 was monitored. A CFS collected from a pure culture of Xcc8004 was also added in TSB10% as a control. We observed a significant (*P <* .05) reduction of Xcc8004 growth in CFS collected from co-cultures of Xcc8004 with *P. lactucae* CFBP 13502 (Fig. 3B). In contrast, no significant difference in Xcc8004 growth was observed following supplementation of the CFS derived from *P. lactucae* CFBP 13502Δ*ltcJ* (Fig. 3B)*.* Complementation of *P. lactucae* CFBP 13502Δ*ltcJ* with a full copy of *ltcJ* partially restored the growth inhibition of Xcc8004 (Fig. 3B).

If iron-chelation by lactuchelin is responsible for the growth reduction of Xcc8004, supplementation of CFS with FeCl_3_ should alleviate the growth inhibition. A gradual increase in Xcc8004 growth was observed following supplementation of CFS from Xcc8004 and *P. lactucae* CFBP 13502 co-culture with a range of FeCl_3_ concentration (from 0.39 μM to 50 μM, Fig. 3C). These results suggest that iron chelation by lactuchelin produced by *P. lactucae* CFBP 13502 is likely involved in the decrease of Xcc8004 growth.

**Figure 4:**
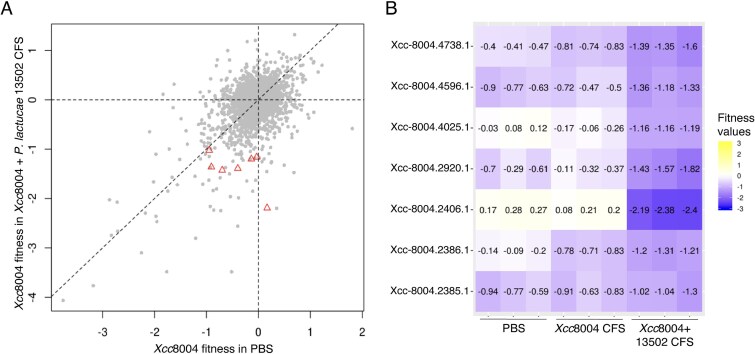
RB-TnSeq screening reveals *Xanthomonas campestris* pv. *campestris* fitness determinants in inhibitory CFS from co-culture of *P. lactucae* CFBP 13502 and Xcc8004. (A) Comparison of gene contribution to fitness in TSB10 supplemented with PBS (control) and in TSB10 supplemented with *P. lactucae* CFBP 13502 and Xcc8004 CFS. Genes with no significant fitness are in gray. Genes found as differentially represented in CFS condition are shown as red triangles. Each dot corresponds to the mean of 3 replicates. (B) Heatmap highlights fitness (gradient color) of the significant Xcc8004 genes in the different conditions.

To explore the impact of the inhibitory CFS on the physiology of Xcc8004 we employed a RB-TnSeq library of Xcc8004 [43]. The abundances of each barcoded mutant were estimated after growth in TSB10% supplemented with (*i*) PBS, (*ii*) CFS of Xcc8004, and (*iii*) CFS of Xcc8004-CFBP 13502. When employing PBS and CFS of Xcc8004 samples as a control group, we identified seven genes with negative fitness values (Fig. 4A and B). However, only one mutant in a gene (Xcc-8004.2406.1 or *mntH)* that encodes a manganese exporter protein was specifically altered (T-test<0.01) in the CFS of Xcc8004-CFBP 13502 in comparison to PBS and CFS from pure cultures of Xcc8004 (Fig. 4B).

To determine whether the growth inhibition of Xcc8004 by lactuchelin observed in vitro could also be found *in planta*, Xcc8004 was either co-inoculated on seeds with *P. lactucae* CFBP 13502 or CFBP 13502Δ*ltcJ.* Under these experimental conditions, population sizes of Xcc on radish seedlings were identical regardless of the CFBP 13502 strain inoculated (Fig. S5). In addition, the CFBP 13502 wt strain and its isogenic lactuchelin-deficient mutant colonized seedlings at similar population sizes (Fig. S5). This indicates that this siderophore is not involved in the transmission phases from seeds to seedlings.

### Identification and structural characterization of lactuchelins *in P. lactucae* CFBP 13502 cell-free supernatant

To identify the putative lipopeptide siderophore, we performed comparative metabolic profiling of CFSs derived from pure cultures of *P. lactucae* CFBP 13502 and Xcc8004 and CFS from the co-culture of *P. lactucae* CFBP 13502 and Xcc8004. Four different organic solvents (AcOEt, BuOH, DCM, and MtBE) were employed for liquid/liquid extraction with H2O. The BuOH-extracted fraction had the same anti-Xcc8004 activity than CFS from coculture of *P. lactucae* CFBP 13502 and Xcc8004 (Fig. S6).

Discriminant analysis of the different BuOH extracts in UPLC-HRMS/MS in negative ionization mode highlighted 14 compounds enriched in the co-culture of *P. lactucae* CFBP 13502 and Xcc8004 (Table 2), potentially involved in bioactivity. Each of them harbors a neutral mass in accordance with a lipopeptide generated by the BGC previously identified (minimal molecular mass 795.64 according to the putative structure of the peptide backbone synthesized by the NRPS identified).

**Table 2:** Metabolites enriched in the cell-free supernatant (CFS) of a coculture of *P. lactucae* CFBP 13502 and Xcc8004, detected in negative ionization mode.

Peak number	Retention time (min)	m/z	Neutral mass	ANOVA (*P*)	Max fold change
1	19.16	515.6610	1033.3366	2.05E-11	11
2	19.23	501.1806	1004.3758	1.60E-09	103
3	20.56	1023.3334	1024.3407	2.22E-16	36
4	23.08	548.1783	1098.3712	3.66E-15	19
5	23.08	1051.3659	1052.3732	4.44E-16	31
6	23.34	542.6866	1087.3878	1.28E-13	8
7	23.58	1121.3526	1122.3599	1.12E-10	13
8	23.70	1077.3797	1078.3870	1.11E-16	49
9	23.90	529.4484	1060.9114	1.08E-12	40 290
10	23.90	548.1962	1098.4083	1.75E-08	12
11	24.23	1088.3928	1089.4001	2.07E-08	10
12	24.41	557.1781	1116.3708	1.59E-12	25
13	24.41	562.1927	1126.4000	5.90E-14	32
14	24.41	1079.3940	1080.4013	1.67E-15	28

The table includes the peak number (Peak number), retention time in minutes (Retention time), observed mass-to-charge ratio (m/z), calculated neutral mass (Neutral mass), statistical significance of enrichment (ANOVA *P* value), and the maximum fold change observed between conditions (Max fold change).

*P. lactucae* CFBP 13502 was then cultivated in M9 minimal medium to improve the production of compounds involved in iron chelation. The production of the metabolites described in Table 2 was increased (Fig. S7) as well as the inhibitory activity of the CFS (Fig. S8). Twenty-six compounds with *m/z* ranging from 951.3821 to 1116.4193 were detected in HRMS analysis in negative mode. They were classified into three series, where mass differences of 2 or 28 between two molecules correspond to an additional unsaturation or an acetyl group, respectively. A Δm/z of 52.9114 was calculated for certain compound pairs between series 1 and 2, indicative of a ferric complex [M - 3H + Fe^3+^], whereas a Δm/z of 23.9581 was observed between compounds of series 1 and 3, corresponding to an aluminum complex [M - 3H + Al^3+^]. Altogether, these findings indicate that series 1 corresponds to the free forms of the siderophores, series 2 to their ferric complexes, and series 3 to their aluminum complexes. Thus, nine siderophores were identified in their free, Fe(III)-bound, or Al(III)-bound forms, differing primarily by the length of their fatty acid chain, ranging from C8 to C16 (Table S5). Furthermore, these nine siderophores are not detected in the chromatograms of the CFS from the non-producing mutant, but they are detected in CFS from the complemented strain although at a lower concentration than the wild-type strain (Fig. S9).

**Figure 5:**
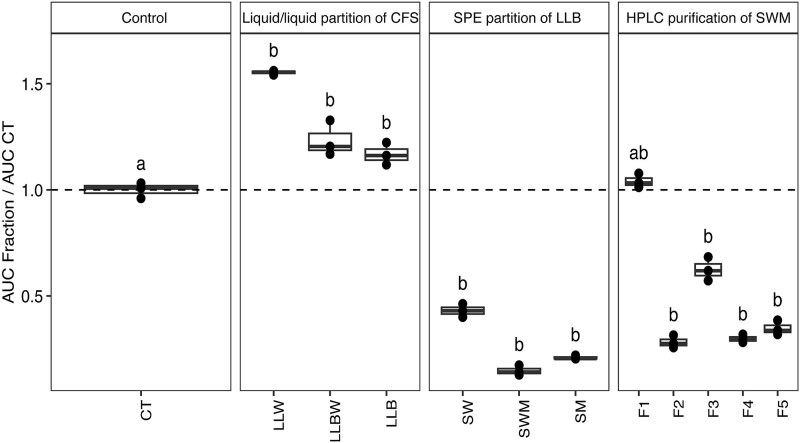
Xcc8004 growth inhibition of the fractions during the purification process of compound 6. Xcc8004 growth was monitored in TSB10% medium supplemented with fractions from CFS of culture of *P. lactucae* CFBP 13502 in iron-depleted M9. Growth inhibition was evaluated by the area under the curve of growth curves (AUC). CT: control, LLW: water extract from liquid/liquid partition, LLBW: BuOH/water emulsion from liquid/liquid partition, LLB: BuOH extract from liquid/liquid partition, SW: water fraction from solid phase extraction (SPE) partition of BuOH extract, SWM: 50/50 water/MeOH (v/v) fraction from SPE partition of BuOH extract, SM: MeOH fraction from SPE partition of BuOH extract, F1, F2, F3, F4, and F5: fractions obtained from purification of SWM on preparative HPLC.

The inhibitory activity of the different fractions obtained during the purification process was evaluated against Xcc8004 (Fig. 5). The fraction F4, with a strong inhibitory activity, contained a purified compound (6*—*Table S5). The LC–MS/MS analysis in negative mode of this compound revealed an iron-complex form with m/z at 1086.4011, corresponding to [M − 3H + Fe^3+^]^−^ of the Fe(III)-(6), which was consistent with a molecular formula of C_43_H_67_FeN_8_O_21_, the associated free form detected in non-supplemented CFS harbor a m/z at 1033.4591 [M-H]^−^, in accordance with a molecular formula of C_43_H_70_N_8_O_21_. The mass fragmentation pattern in positive mode of the isolated compound (6) confirmed the partial amino acid sequence: [fatty acid]-[ꞵOHAsp]-[Ser]-[Ser]-[Ser] (Fig. S10). Additional fragments of m/z 417.16 and 286.14, corresponding to a neutral loss of 131.02 validated the presence of a second ꞵ-OH-Asp moiety. Iron complexed with compound (6) was removed by treatment with 8-hydroxyquinoline and the free form of compound (6) was complexed with gallium for NMR analysis.

**Figure 6:**
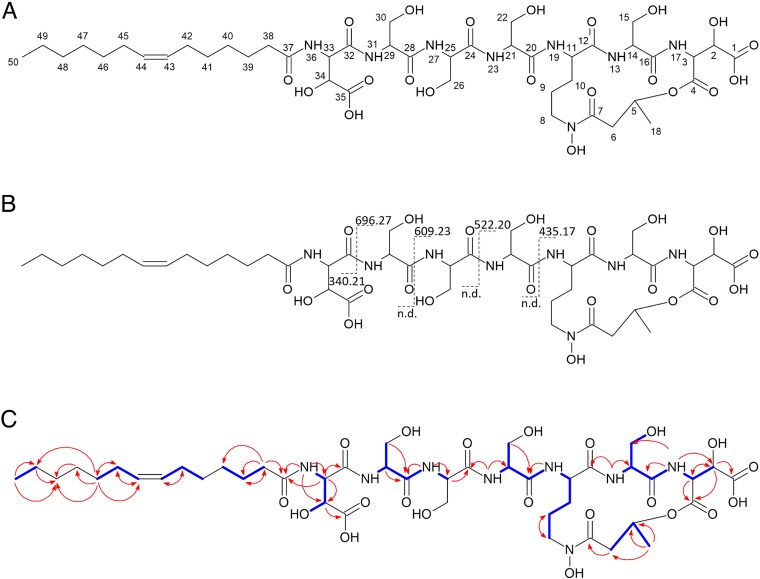
Structural characterization of lactuchelin A. (A) Structure of lactuchelin A. (B) Fragmentation pattern of lactuchelin A in MS/MS experiment, dashed lines represent the characteristic “y” and “b” ions. nd: not detected (C) key ^1^H − ^1^H COSY (blue line) and ^1^H-^13^C HMBC (red arrow) correlations of lactuchelin A.

Structural characterization of Ga(III)-(6) was achieved using ^1^H, ^13^C, COSY, HSQC, and HMBC experiments (Table S6 and Fig. 6), as well as genetic predictions and MS/MS data. Initial analysis of the ^1^H and COSY spectrum revealed the presence of a lipopeptide containing eight spin systems. This included the four canonical amino acid Ser and three non-canonical amino acids, two ꞵ-OH-Asp and one ornithine (Orn). A final spin system was identified as a 3-hydroxybutanoic acid (Hbu). A tetradecenoic acid moiety was identified by ^1^H and ^13^C NMR and confirmed by MS/MS analysis. HMBC and COSY correlations from the amide protons to adjacent carbonyl carbons and alpha-protons identified the linear peptide sequence as [ꞵOHAsp]-[Ser]-[Ser]-[Ser]-[Orn]-[Ser]-[ꞵOHAsp]. Due to the absence of correlations between Hbu moiety and other spins systems especially between H-5 of the Hbu moiety and the ɑ-carbonyl C-3 of ꞵOHAsp and carbonyl C-7 of Hbu moiety with the H-8 of Orn, the cyclization of the peptide core was confirmed by comparison with the literature data in particular with imaqobactin [21]. This confirmed the presence of a cyclic depsipeptide linked between Orn and ꞵOHAsp residues via Hbu. Finally, an HMBC correlation between NH-36 and C-37 of the tetradecenoic acid moiety placed it on the NH-36 position coupled via an amide linkage. The location of unsaturation was determined by the analysis of all HMBC and COSY correlations. Unfortunately, the geometry of unsaturation was impossible to resolve due to the impossibility to distinguish H-43 and H-44 both to δH 5.32. The isolated compound (6) was named lactuchelin A.

## Discussion

By screening a collection of seed-borne bacterial strains for antagonistic properties towards the seed-transmitted pathogen *X. campestris* pv. *campestris* 8004, we identified a cell-free supernatant (CFS) that decreased Xcc8004 growth of ~40%. The antagonistic activity of this CFS was observed during co-culture of the producer strain *P. lactucae* CFBP 13502 with four specific bacterial strains tested in this work including Xcc8004. The compounds involved in the growth inhibition of Xcc8004 are a new class of lipopeptide siderophore that we named lactuchelins.

Lactuchelins are newly described hydroxamate siderophores featuring a cyclic depsipeptide core composed of serine, β-hydroxy-aspartate, and N-hydroxyornithine, linked *via* ester and amide bonds, with an additional 3-hydroxybutanoic acid moiety. This type of cyclization has only been reported so far in imaqobactin [21]. According to metabolomics we were able to identify nine compounds, detected in three different forms: free, complexed with iron or aluminium. Due to low abundance of these compounds, only one—lactuchelin A—could be fully characterized. The production of lipopeptide siderophores in suites differing by the fatty acid tails is a well-known process, primarily observed in marine environments [55]. However, terrestrial examples have also been reported, such as the cupriachelins from the bioplastic producer *Cupriavidus necator* [24] and serobactins from the grass endophyte *H. seropedicae* [25]*.* The combination of seven amino acid cores with a long fatty acyl chain results in amphiphilic compounds excreted in microbial supernatants. The variation of fatty acid chain length (ranging from C8 to C16) within the lactuchelins series enhances the partitioning of these siderophores into cell membranes. This structural diversity likely improves not only iron uptake [19] but also the acquisition of other trivalent metals, such as aluminium (III), in *P. lactucae*, as evidenced by the detection of Al(III) complexes.

Although HRMS analysis indicated that lactuchelins can complex ferric iron and aluminium, several lines of evidence indicated that chelation of iron is involved in the growth inhibition of Xcc8004. First FeCl_3_ supplementation of the CFS completely relieved growth inhibition. Second, RB-TnSeq experiments have highlighted a decrease of fitness of *mntH* deficient mutants in Xcc8004 following supplementation with CFS from the coculture of Xcc8004 and *P. lactucae* CFBP 13502. In *Neisseria meningitidis*, *mntH* encodes a manganese exporter protein that regulates the intracellular ratio of Mn/Fe conferring protection against manganese toxicity at low iron concentration [56]. The decrease of fitness in *mntH* mutants of Xcc8004 can therefore be explained by the lower bioavailability of iron (due to iron chelation by lactuchelin), which results in an increase in the intracellular ratio of Mn/Fe within Xcc8004 cells. In the absence of a functional manganese exporter, overaccumulation of manganese affects Xcc8004 growth. Third, other *Xanthomonas* species are sensitive to iron deprivation by siderophores. For instance pyoverdine productions by *P. putida* KT2440 can inhibit the growth of *Xanthomonas fragariae* [57]. Finally, the growth inhibition of a large number of strains (n = 27) following CFS supplementation of the co-culture of Xcc8004 and *P. lactucae* CFBP 13502 supports the chelation of an essential element for bacterial growth. The three strains that were not impacted by this CFS corresponded to *P. lactucae* CFBP 13502, *P. coleopterorum* CFBP 13575, and *P. asgharzadehiana* CFBP 13506. The two first strains (CFBP 13502 and CFBP 13575) possessed the BGC of lactuchelins that include the TonB-dependent transporter LtcK potentially involved in lactuchelins uptake. Although *P. asgharzadehiana* CFBP 13506 does not have this BGC, an ortholog of LtcK is encoded in its genome sequence. Therefore, this strain can potentially utilize lactuchelins produced by *P. lactucae* CFBP 13502 *via* xenosiderophore utilization, a strategy called siderophore piracy [58] and used for siderotyping of *Pseudomonas* strains in taxonomic studies [59]. In line with this hypothesis, it is striking to note that *P. lactucae* is the closest species from *P. asgharzadehiana.*

In comparison to pyoverdine, the distribution of lactuchelin biosynthesis genes within *Pseudomonas* is much less important, representing 0.1% of the genome sequences available versus 97% for pyoverdine [16]. Lactuchelins BGC is found in all sequenced strains of *P. lactucae* [29]*, P. paraveronii* [60], *P. versuta* [61]*, P. paraversuta* [62], *P. californiensis* [63], *P. coleopterorum* [64], and *P. typographi* [65] species. These lactuchelin-producing strains do not possess pyoverdine BGC. This “mutual exclusion” between siderophores is not frequent. Indeed, most pyoverdine-producers strains can usually synthesize other siderophores (known as secondary siderophores because of their lower affinity for ferric iron) including the lipopeptide siderophores ornicorrugatin [66] and histicorrugatin [18]. The absence of pyoverdine BGC in lactuchelin-producers is probably due to the fact that producing these two siderophores is too costly to maintain. Lactuchelin production could reflect an iron acquisition strategy i.e. not solely based on the diffusion of siderophores in the external environment (as is the case with pyoverdines). Indeed the amphiphilic nature of lactuchelin could limit siderophore diffusion through membrane association [20]. As a result, moderate siderophore secretion could then limit their use by individuals other than the producers [67].

The induction of lactuchelin production by *P. lactucae* CFBP 13502 is specifically triggered during coculture with three *Lysobacteraceae* strains (Xcc8004, *S. rhizophila* CFBP 13503, and CFBP 13529) and one strain of *B. simplex* (CFBP 13531). This raises questions about the signals involved in this induction. The simplest hypothesis would be a decrease in iron concentration in the extracellular environment, which would lead to the transcription of *pvdS*. PvdS is an RNA polymerase sigma factor, which activates transcription of genes for the biosynthesis or the uptake of siderophores. It was originally described as activating expression of pyoverdine biosynthetic genes [68]. Although *P. lactucae* CFBP 13502 does not possess the pyoverdine biosynthetic gene cluster, it appears that PvdS may be also involved in activating the expression of lactuchelin biosynthetic genes. This would explain why a mutation in *pvdS* abolishes lactuchelin production in CFBP13502. However, if iron concentration is the factor involved in this modulation, it is difficult to understand why only a few strains (four of thirty) trigger the production of lactuchelin. An alternative assumption would be that this induction may result from other signaling molecules. Previous studies have demonstrated that siderophore production can be regulated by various factors, such as quorum sensing [69], ions [70], and primary metabolites. For example, histidine represses the histidine utilization repressor HutC, thereby inducing pyoverdine synthesis in *P. fluorescens* SBW25, independently of iron limitation [71].

Regardless of the nature of the signals triggering lactuchelin synthesis, it appears that these siderophores are not produced during the transition from seed to seedling. Further investigation of the interactions between Xcc8004 and *P. lactucae* CFBP 13502 is necessary to better understand the mechanisms driving its induction. This knowledge is particularly important if *P. lactucae* CFBP 13502 is to be used as a biocontrol agent in seed treatment.

## Supplementary Material

supplementary_materials_wrag003

## Data Availability

The FASTQ reads used for RNASeq analyses are available in the Sequence Read Archive under the accession number PRJNA787065. All scripts and data sets employed in this work are available from INRAE GitLab https://forge.inrae.fr/irhs-emersys/lactuchelins. MS raw data are available at recherche.data.gouv.fr (https://doi.org/10.57745/CFU7FS).
