# Supplementary material for: Lactuchelins represent lipopeptide siderophores produced by Pseudomonas lactucae that inhibit Xanthomonas campestris
Source: ISME J. 2026 Jan 16;20(1):wrag003. doi: 10.1093/ismejo/wrag003 (PMC12904278; doi:10.1093/ismejo/wrag003)
Supplement: supplementary_materials_wrag003 [file supplementary_materials_wrag003.zip › Supplementary_Methods_v2.pdf]

Supplementary Methods

Reassessment of the taxonomic affiliation of CFBP 13502

Since the first genome-based identification of CFBP 13502, many novel *Pseudomonas* species have been proposed, requiring the reassessment of CFBP 13502 species identity. Calculation of digital DNA-DNA hybridizations to identify the closest type strain genome was performed using the Type (Strain) Genome Server (TYGS) [1]. JSpeciesWS web server [2] and EzBioCloud [3] were used to calculate ANib and orthoANIu respectively with the closest type strain genomes identified. Results were compared to the threshold for species delineation.

DNA Extraction (Genome sequencing and annotation)

A cell pellet was resuspended in PBS to obtain OD_{600} 2.0. One ml of OD_{600} 2.0 culture was centrifuged, decanted, then resuspended in 300 µL TE buffer with 2.5 µL lysozyme (5 $mg.ml^{-1}$) and 1 µL RNaseA (5 $mg.ml^{-1}$). The solution was incubated at 37°C for 45 min. Cells were lysed by adding 300 µL of MasterPure 2x Tissue and Cell Lysis Solution and 1 µL of Proteinase K (50 $mg.ml^{-1}$). Samples were incubated at 65°C for 15 min then placed on ice for 5 min. DNA was further purified using phenol-chloroform extraction.

Total RNA extraction (RNA-Seq experiments)

A 2-ml suspension of co-culture after 6 h growth was centrifuged 30 s at 14,000 G. The pellet was resuspended in 600 µl of hot (65°C) Trizol (Invitrogen, Cergy Pontoise, France) and the mixture was shaken at 65°C for 5 min. After 5 min at room temperature, the cell debris was removed by centrifugation (2,500 G, 5 min at 15°C) and the supernatant was transferred to a fresh tube. Chloroform (120 µl) was added to 600 µl of the supernatant and the mixture was vortexed vigorously 30 s. After 2 min at room temperature, the phases were separated by centrifugation at 12,000 G for 15 min at 4°C and the upper phase (300 µl) was removed to a fresh tube. Isopropyl alcohol (250 µl) was added and the mixture was incubated at room temperature for 10 min. The precipitated RNA was collected by centrifugation at 12,000 G for 20 min at 4°C. The supernatant was removed and the pellet was washed with 500 µl of 75% ethanol. A new pellet of RNA was collected by centrifugation at 7,500 G for 5 min at 4°C and was air dried to near completion. The RNA was dissolved in 89 µl of sterile double-distilled H_2O, and the preparation was treated with 1 µl (2 U) of DNase (Ambion Applied Biosystems, Courtaboeuf, France) to remove contaminating DNA during 1 h at 37°C. The preparation was heated 10 min at 75°C to inactivate the DNAse and RNA was ethanol precipitated and dissolved in 50 µl of double-distilled H_2O.

TEDA Cloning

Briefly, pEX18Tc was digested with KpnI (New England Biolabs) followed by shrimp alkaline phosphatase treatment (New England Biolabs). *ltcJ* flanking regions were PCR-amplified from CFBP 13502 with the AccuPrime Taq DNA Polymerase High Fidelity (Invitrogen) and the primer pairs listed in Table S2 (Upe403F/Upe807R_1510RC for upstream region and Dn1510F/Dne1920R for downstream region). The linearized and dephosphorylated pEX18Tc

vector and PCR products were purified using the NucleoSpin Gel and PCR Clean-up kit (Macherey-Nagel). TEDA reaction was then carried out by mixing 100 ng of pEX18Tc with the corresponding PCR products at a molar ratio of 1:4:4. One hundred µl of *E. coli* MFDpir were transformed with ¼ of TEDA reaction mix (5 µl) using the Inoue transformation procedure [4] modified by [5]. Amplicon insertions were validated by colony PCR with the primer pair M13F/M13R and primers pairs listed in Table S2. Plasmids (pEX18Tc-Δ*ltcJ*) were extracted with the NucleoSpin plasmid kit (Macherey-Nagel) by using low copy extraction protocol, and insertion regions were verified by sequencing.

Preparation of CAS Agar medium

Briefly, 850 ml of MM9 medium (KH2PO4 0.026 mM, NaCl 0.1 mM, and NH4Cl 0.22 mM) buffered with 32.24 g of PIPES was autoclaved with 15 g of agar. Thirty milliliters of sterile iron-depleted casamino acid solution (0.11 $g.l^{-1}$) and 10 ml of sterile 20% glucose solution were added to the agar MM9. A blue dye solution (chromazurol S (CAS) 1 mM, FeCl3-6H2O 0.09 mM, and HDTMA 2 mM) was finally slowly added to the mixture and plates were poured with this CAS agar medium.

Pooled mutant fitness assays (Genome-wide mutant fitness assays)

The Xcc8004 mutant library was revived in MOKA rich medium (Yeast Extract 4 $g.l^{-1}$, Casamino acids 8 $g.l^{-1}$, K2HPO4 2 $g.l^{-1}$, and MgSO4.7H2O 0.3 $g.l^{-1}$) supplemented with rifampicin, 50 $µg.ml^{-1}$; kanamycin: 50 $µg.ml^{-1}$; tetracycline: 5 $µg.ml^{-1}$. Culture was left to grow at 28°C under constant agitation (150 rpm) until reaching mid-log phase. Samples of the culture of Xcc8004 pooled mutants were collected as the “Time0” controls. The remaining culture was pelleted and washed two times then resuspended in 10 mM MgSO4.

DNA Extraction (Genome-wide mutant fitness assays)

Two hundred nanograms of DNA were amplified using Q5 Polymerase (NEB) and BarSeq primers [6]. PCR cycling conditions were as follows: denaturation at 98°C (4 min), 25 cycles at 98°C (30 sec), 55°C (30 sec) and 72°C (30 sec), and a final elongation at 72°C for 5 min. Amplicons were purified with magnetic beads and pooled in equimolar concentration. Concentration of the pool was monitored with quantitative PCR (KAPA Library Quantification Kit, Roche) and sequenced on v3 150 Illumina cartridge (Illumina, San Diego, CA).

Pipeline for acquisition of mutant fitness scores (Genome-wide mutant fitness assays)

Mutant fitness scores were calculated as the normalized log2 ratio of the abundance of its barcode in the library under our experimental conditions versus the “Time0”. Each mutant is associated with a unique barcode; thus the reads of a certain barcode characterize the abundance of the mutant inside the library. Gene fitness is calculated as the weighted mean of the fitness of each individual mutant. Gene fitness were then normalized to correct variation in copy number along the chromosome, the running median along the chromosome is zero. For each fitness score, we performed a t-like test.

Metabolomic analysis procedures (UPLC-HRMS/MS)

A sample called “Mix” containing an equivalent mixture of the 4 fractions at 100 $µg.ml^{-1}$ was used to align all the chromatograms obtained and to check the statistical analysis by PCA.

Each sample was injected 5 times in both positive and negative modes. The analysis was performed using a Waters Acquity UPLC H-Class Series system. Chromatographic separation was carried out on an Acquity HSS T3 column (100 mm x 2.1 mm, 1.8 µm) at 25°C. The mobile phase consisted of solvent A (0.1% formic acid in water) and solvent B (0.1% formic acid in acetonitrile), with gradient from 0 to 35% of B in 20 min then 35% to 100% of B in 5 min held for 3 min. The flow rate was set at 300 µl.min^{-1}. The injection volume was 1 µl.

Mass spectrometry was performed on a Waters Xevo G2-XS system. The analyses were performed as two separated methods, one operated in positive electrospray ionization (ESI) mode and one in negative ESI mode. Mass data were acquired in MSe Continuum sensitivity mode with a mass range from m/z 50 to 2000 with a scan rate of 2 s. The tuning parameters (positive and negative) were : capillary voltage 500 V, sampling cone 40 V, cone voltage 80 V, source temperature 120°C, desolvation temperature 500°C, desolvation gas flow 100 l.h^{-1} and cone gas flow 1000 l.h^{-1}. The low collision energy was set at 6 V and the high collision energy was a ramp from 15 to 40 V. The collision pressure of argon was constant at 6.5E^{-3} mbar. Mass correction was performed with leucine enkephalin solution at 200 pg.ml^{-1}, measured at 30 s intervals throughout each injection, at a flow rate of 5 µl.min^{-1}. The ions monitored were m/z 556.2766 and 554.2620 in ESI+ and ESI-, respectively. The data were then processed with the Progenesis QI software. Potential markers of interest were extracted from S-plots constructed following OPLS-DA, and markers were chosen based on their contribution to the variation and correlation within the data set.

Replacement of iron with gallium

For the following procedures, all work was carried out in iron-free glassware. F4 fraction (13.6 mg) was dissolved in 80 ml of H_2O, added to 50 ml of a 8-hydroxyquinoline solution in MeOH (290 mg, 1 mM), and stirred at room temperature for 24 h. The MeOH was then removed in vacuo at 40°C and the remaining aqueous solution was extracted with CH_2Cl_2 (5 × 40 ml). The aqueous layer was mixed with a solution of Ga2(SO4)3 (30 mg, 35 µmol) in sulfuric acid (0.1 N) and the mixture was allowed to mix for 30 min at room temperature. After adjustment of the pH at 7, the solution was freeze-dried.

SWM fractionation with preparative HPLC

SWM fraction was injected on a preparative HPLC Shimadzu LC-20AP solvent delivery system coupled to an SPD-UV detector and an FRC-10A fraction collector using LabSolutions software and a Thermo Fisher Scientific Hypersil Gold PFP column (150 x 20 mm; 5 µm; 175 Å). The flow rate was set at 18.9 ml.min^{-1} with a gradient of (A) water + 0.1% of formic acid and (B) MeCN + 0.1% formic acid (20% to 60% B in 10 min, 60% to 100% B in 2 min, then 100% B for 6 min) and UV detection was set at 190 nm.

References

1. Meier-Kolthoff JP, Göker M. TYGS is an automated high-throughput platform for state-of-the-art genome-based taxonomy. *Nat Commun* 2019; **10**: 2182. https://doi.org/10.1038/s41467-019-10210-3

2. Richter M et al. JSpeciesWS: a web server for prokaryotic species circumscription based on pairwise genome comparison. *Bioinformatics* 2016; **32**: 929–931. https://doi.org/10.1093/bioinformatics/btv681

3. Yoon S-H et al. Introducing EzBioCloud: a taxonomically united database of 16S rRNA gene sequences and whole-genome assemblies. *Int J Syst Evol Microbiol* 2017; **67**: 1613–1617. https://doi.org/10.1099/ijsem.0.001755

4. Sambrook J, Russell DW. The Inoue method for preparation and transformation of competent *E. Coli* : “Ultra-competent” cells. *Cold Spring Harb Protoc* 2006; prot3944. https://doi.org/10.1101/pdb.prot3944

5. Xia Y et al. T5 exonuclease-dependent assembly offers a low-cost method for efficient cloning and site-directed mutagenesis. *Nucleic Acids Res* 2019; **47**: e15–e15. https://doi.org/10.1093/nar/gky1169

6. Wetmore KM et al. Rapid quantification of mutant fitness in diverse bacteria by sequencing randomly bar-coded transposons. *mBio* 2015;**6** : e00306-15. https://doi.org/10.1128/mBio.00306-15

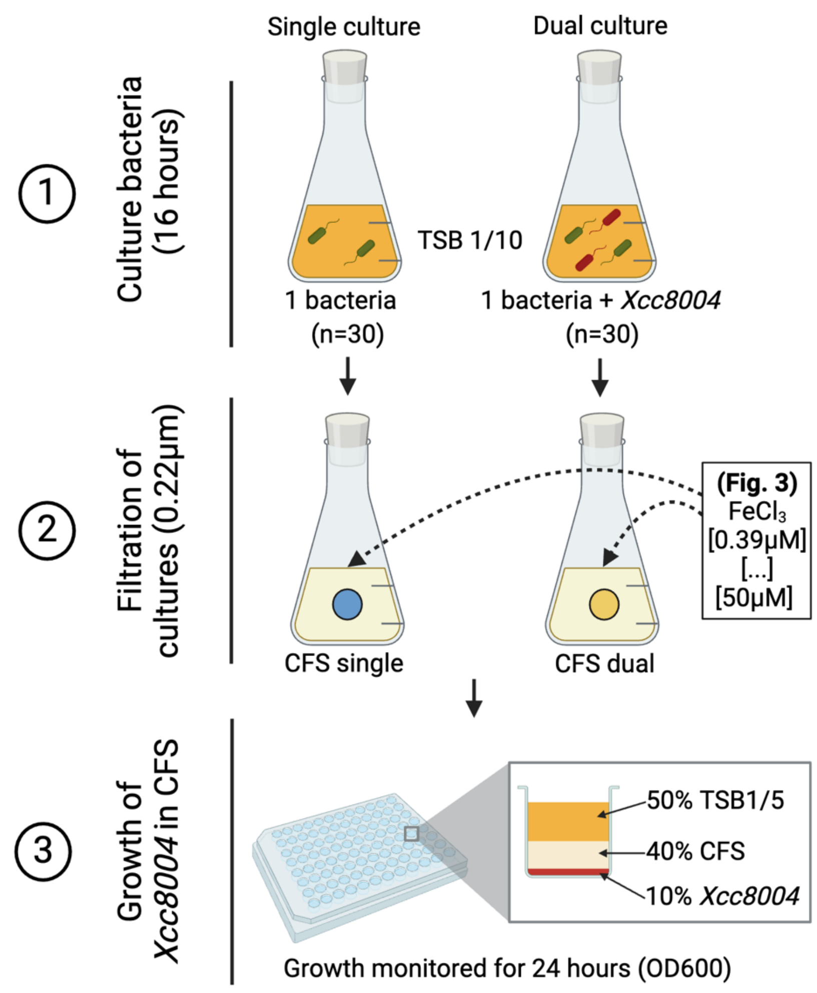

Figure S1: Schematic overview of the cell-free supernatant (CFS) experiments. 1. Bacterial strains were cultured for 16 h, either in monoculture (left) or co-cultured with Xcc8004 (right). **2.** Cultures were filtered through 0.22 µm to obtain the cell-free supernatants (CFSs). Dashed arrows indicate $FeCl_3$ supplementation as shown in Figure 3C. **3.** CFSs were supplemented with TSB1/5 and Xcc8004 to assess the impact of the supernatants on Xcc8004 growth. Figure was created in Biorender.

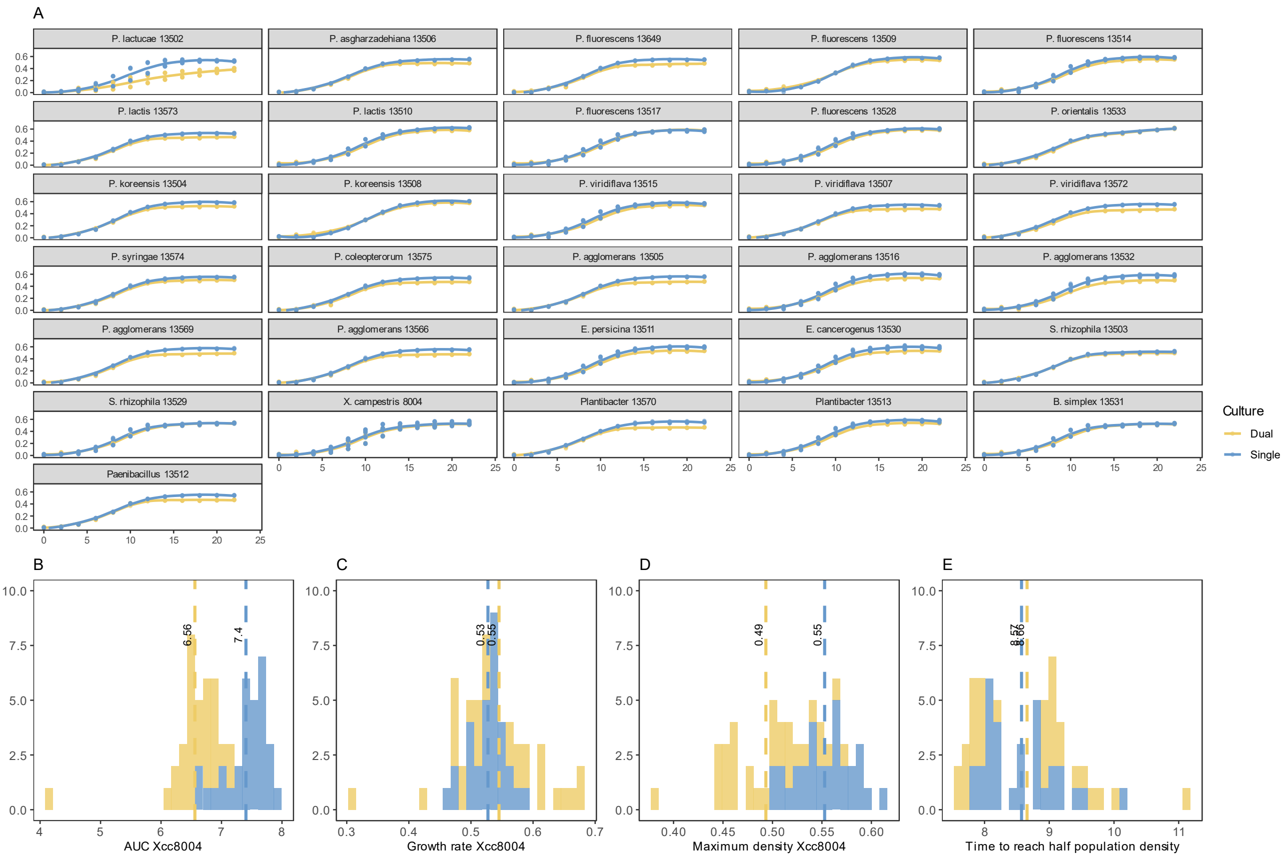

Figure S2: Xcc8004 growth following supplementation with cell-free supernatants (CFSs). Xcc8004 growth was monitored (OD_{600}) over time (24 h) in TSB10% medium supplemented with single (blue) or dual CFSs (yellow). Xcc8004 growth curves are presented (**A**) along with the following parameters: area under the curve (**B**), growth rate in h^{-1} (**C**), maximum density as estimated with OD_{600nm} (**D**), and time (hours) to reach half of the population density (**E**).

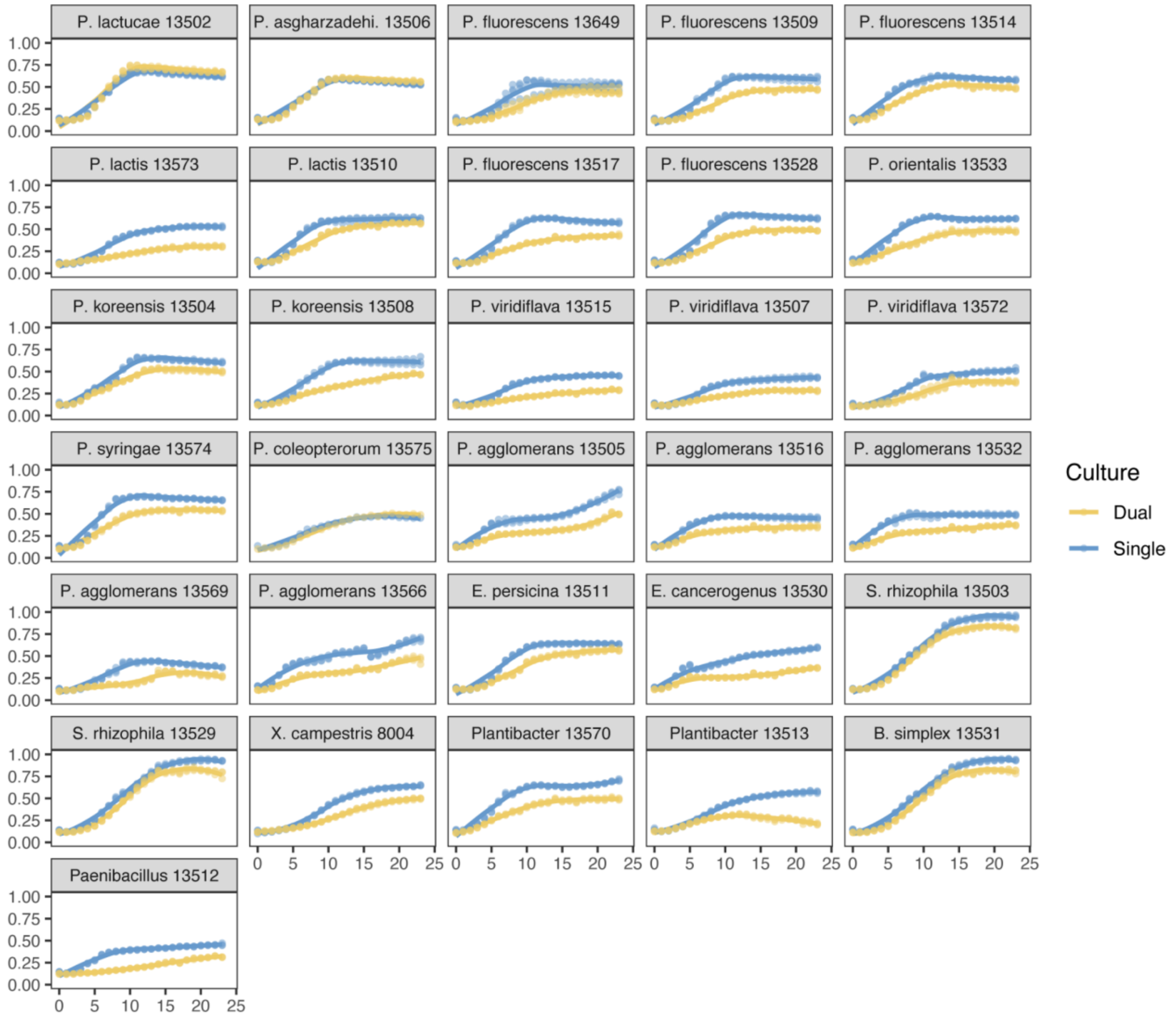

Figure S3: CFS of CFBP 13502 and Xcc8004 inhibited the growth of a wide range of bacterial taxa. Thirthy-one bacterial strains growth (panels) were measured for 24 h in TSB10% supplemented with CFS of CFBP 13502 (single - blue) or CFS of CFBP 13502+Xcc8004 (dual - yellow), using spectrophotometer. Three replicates were performed for each condition.

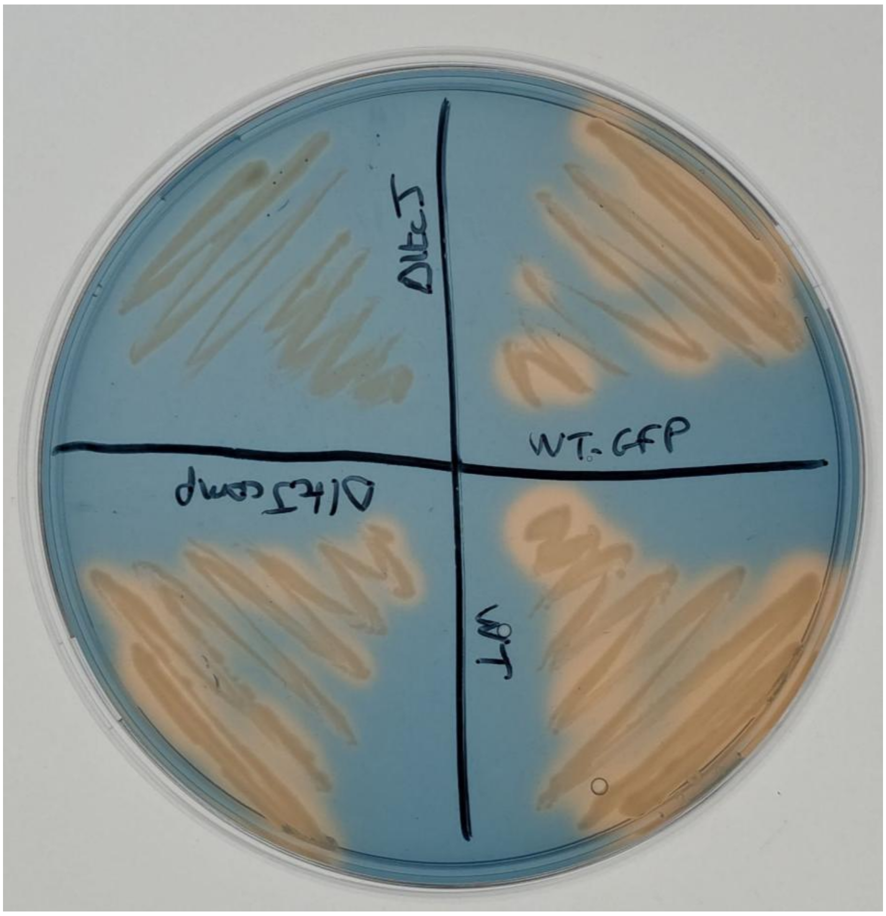

Figure S4: *ltcJ* is involved in bacterial siderophore production. CFBP 13502 strains and mutants on CAS agar plate, orange halo showed a production of siderophores. WT: CFBP 13502 wild-type, WT-GFP: CFBP 13502 GFP-complemented, Δ*ltcJ*: CFBP 13502Δ*ltcJ*, and Δ*ltcJ*comp: CFBP 13502Δ*ltcJ att*Tn7::*ltcJ*.

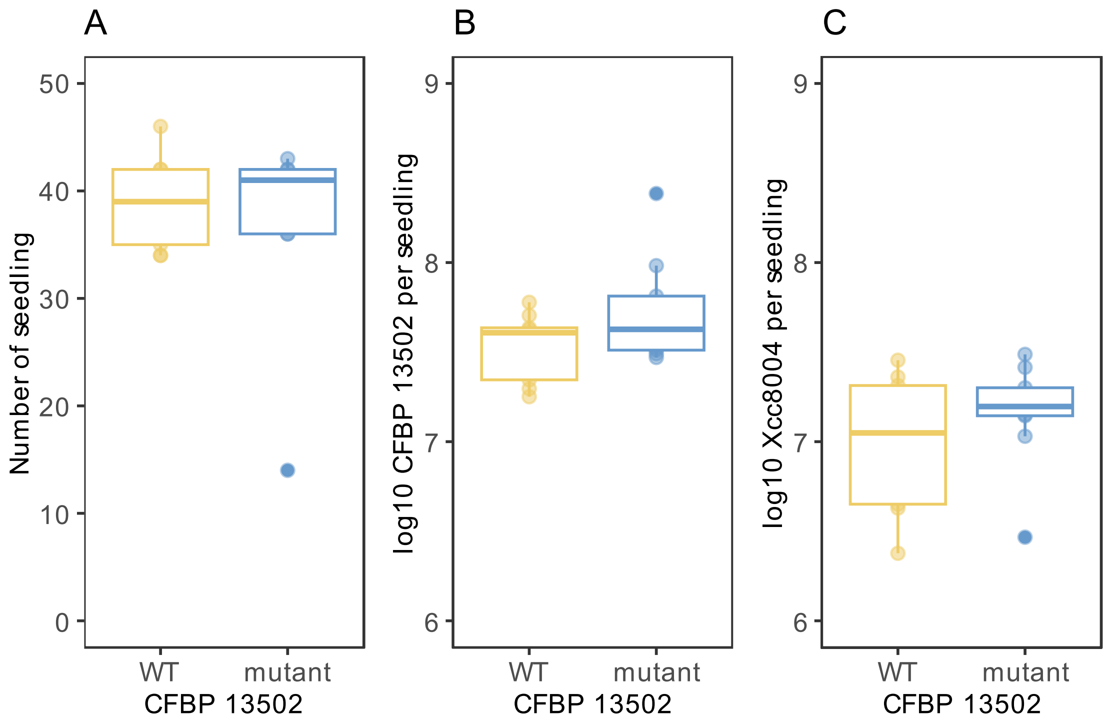

Figure S5: Seed inoculation of Xcc8004 with *P. lactucae* CFBP 13502 wild-type or its isogenic lactuchelin-deficient mutant (Δ*ltcJ*). The number of seedlings (60 hours post-inoculation) collected (**A**), the number of colony-forming units of CFBP 13502 (**B**) or Xcc8004 per seedling (**C**) are reported. Three independent biological replicates, each consisting of three technical replicates, were performed. No statistical difference in seedling number or CFU per seedling were observed (t-test, *P* value<0.05).

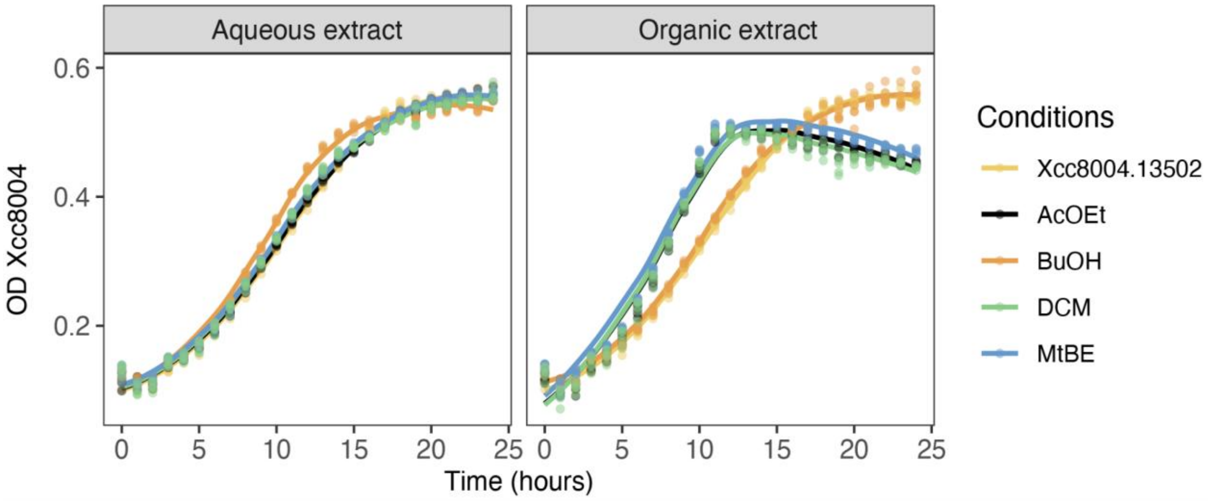

Figure S6: BuOH extracts inhibits Xcc8004 growth. Xcc8004 growth monitored (OD_{600}) over time (24 h) in TSB10% medium supplemented with fractions ethyl acetate (AcOEt), butanol (BuOH), dichloromethane (DCM) and methyl *tert*-butyl ether (MtBE) obtained from CFS of Xcc8004 and CFBP 13502.

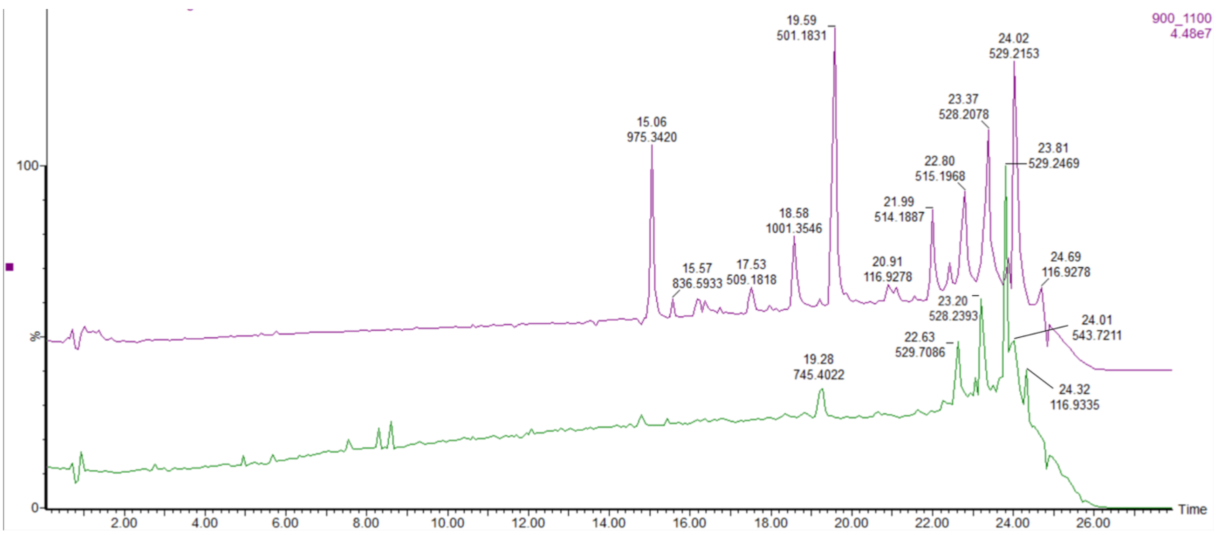

Figure S7: Extracted ion chromatograms of m/z between 900 and 1100, showing an increase of lactuchelins production in M9 medium. Green trace, bottom: CFS of coculture of CFBP 13502 and Xcc8004; purple trace, up: single culture of CFBP 13502 in M9 medium supplemented with glucose (20 mM).

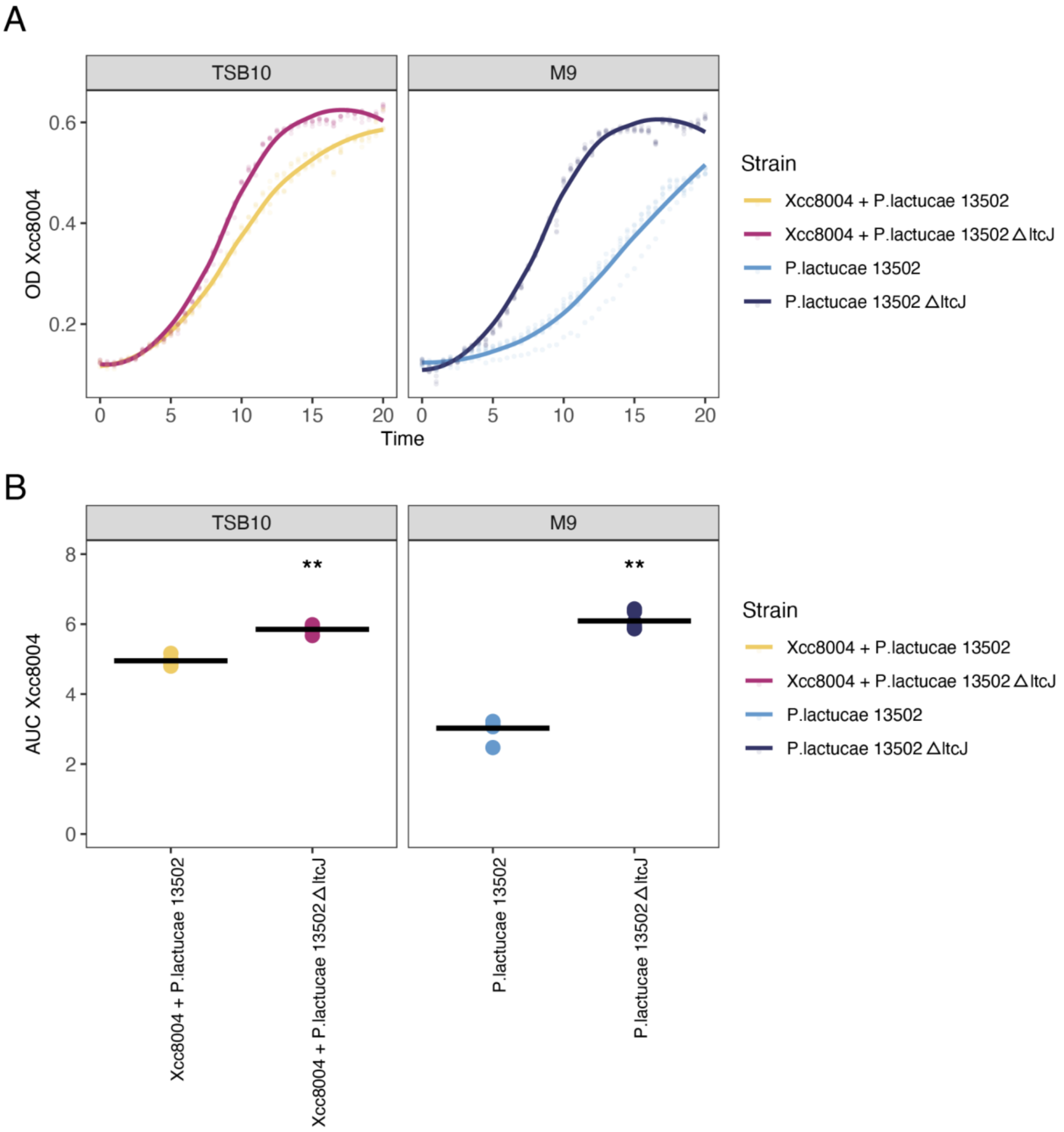

Figure S8: Culture of CFBP 13502 in M9 medium improves the anti-Xcc8004 effect of the CFS. Xcc8004 growth monitored in CFSs from co-cultures of Xcc8004 and CFBP 13502 (yellow), CFBP 13502Δ*ltcJ* (purple), CFSs from single culture of CFBP 13502 in M9 medium (light blue), and CFBP 13502Δ*ltcJ* in M9 medium (dark blue). **A.** Xcc8004 growth curves. **B.** AUC of Xcc8004 in the 4 different culture conditions. Stars denote statistically significant differences (Wilcoxon-test, ** *P* value < 0.01).

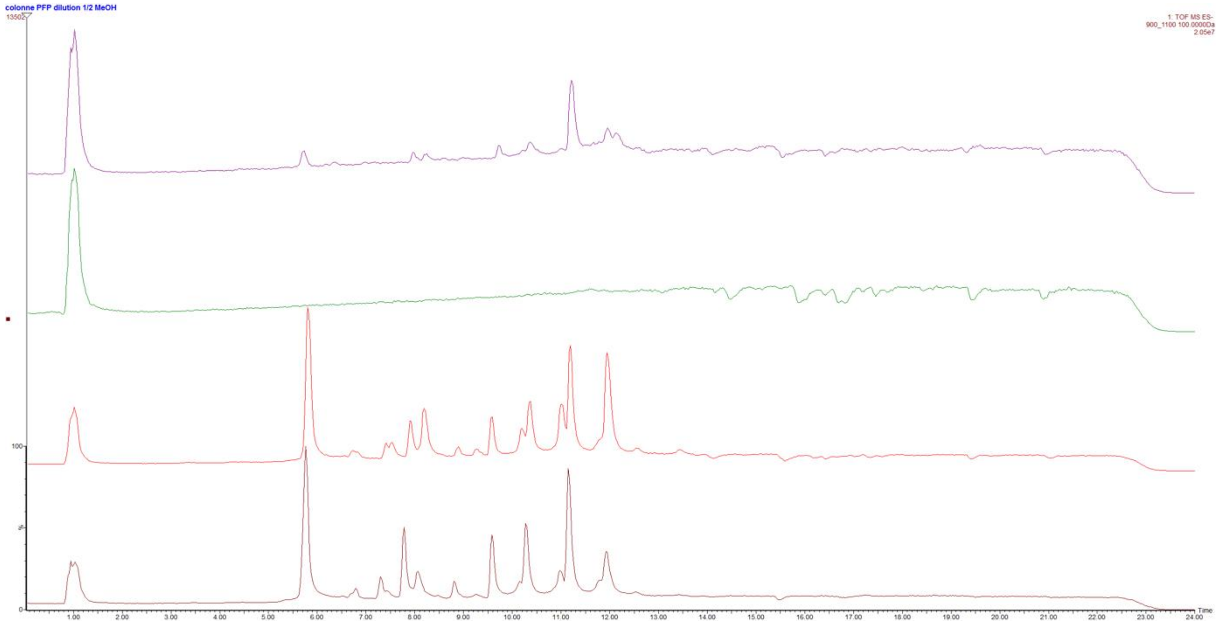

Figure S9: Extracted ion chromatograms of m/z between 900 and 1100, showing the disappearance of lactuchelins production in deletion mutant (green trace) and the partial restoration of the production after complementation with *ltcJ* (purple trace). Brown trace, bottom: CFS of culture of CFBP 13502 WT; red trace: CFS of culture of CFBP 13502-GFP; green trace: CFS of culture of CFBP 13502-Δ*ltcJ* and purple trace, up: CFS of culture of CFBP 13502Δ*ltcJ att*Tn7*::ltcJ*.

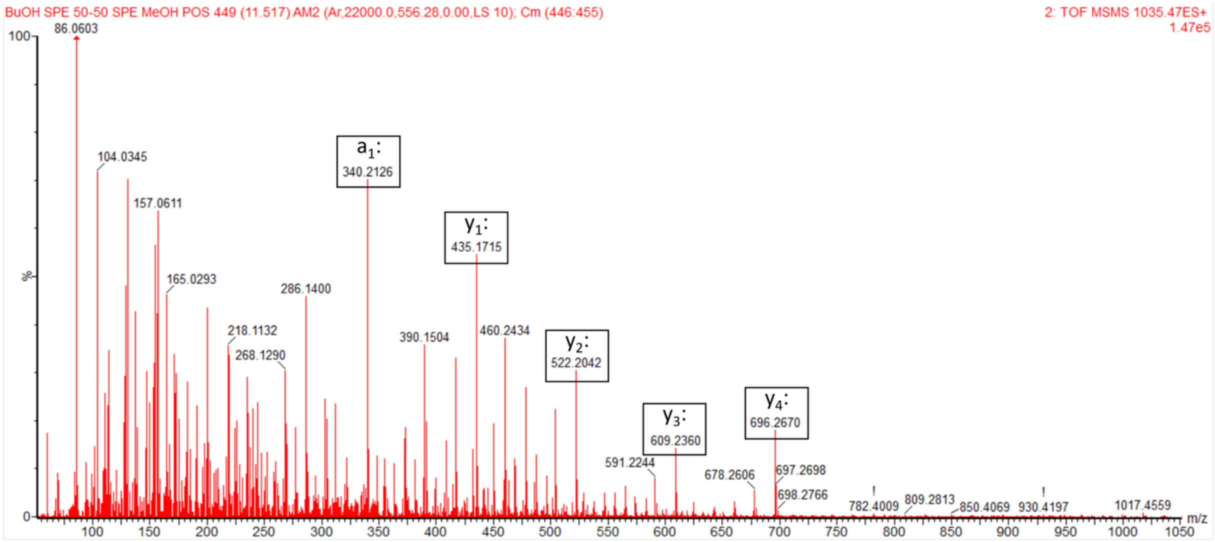

Figure S10: Fragmentation spectra of compound (1) in positive mode m/z 1035.4727
