# Supplementary material for: Lactuchelins represent lipopeptide siderophores produced by Pseudomonas lactucae that inhibit Xanthomonas campestris
Source: ISME J. 2026 Jan 16;20(1):wrag003. doi: 10.1093/ismejo/wrag003 (PMC12904278; doi:10.1093/ismejo/wrag003)
Supplement: supplementary_materials_wrag003 [file supplementary_materials_wrag003.zip › Supplementary_Table.docx]

**Supplementary materials**

**Table S1: Bacterial strains used in this study.**

**Table S2: Primers used in this study.**

**Table S3: Differentially expressed genes of *P. lactucae* CFBP 13502.**

**Table S4: Distribution of the lactuchelin BGC within *Pseudomonas* strains.**

**Table S5: Compounds corresponding to lactuchelins detected in CFS of CFBP 13502 and masses of their different forms (free, Fe3+, Al3+).**

**Table S6: ^1^H NMR (600 MHz) and ^13^C NMR (150 MHz) spectroscopic data of Ga(III)-(1) in DMSO-d_6_ (δ in ppm, J in Hz).**
